# Aziridine Ring Opening as Regio- and Stereoselective Access to *C*-Glycosyl-Aminoethyl Sulfide Derivatives

**DOI:** 10.3390/molecules27061764

**Published:** 2022-03-08

**Authors:** Aleksandra Tracz, Martyna Malinowska, Stanisław Leśniak, Anna Zawisza

**Affiliations:** Department of Organic and Applied Chemistry, University of Łódź, 91-403 Łódź, Poland; aleksandra.tracz@chemia.uni.lodz.pl (A.T.); martyna.malinowska@chemia.uni.lodz.pl (M.M.); stanislaw.lesniak@chemia.uni.lodz.pl (S.L.)

**Keywords:** glycals, aziridine, sulfides

## Abstract

A short synthetic route to stereoselective access to *C*-glycosyl-aminoethyl sulfide derivatives has been developed through the reaction of tributhyltin derivatives of glycals with aziridinecarboaldehyde and the regioselective ring opening of a chiral aziridine with thiophenol. The absolute configurations of the resulting diastereoisomers were determined by 1H NMR spectroscopy.

## 1. Introduction

Intensively developed in recent years, asymmetric synthesis has proved to be a powerful tool in the synthesis of drugs and natural products as well as in the transformation of readily available simple compounds into chiral building blocks used for the synthesis of more complex connections [1,2,3]. Although sugars are the most readily available raw materials, they were long considered of little use due to the presence of polar functional groups. However, it turns out th–t the structure is in fact a great advantage, enabling wide-ranging possibilities of modification, making them very useful synthetic tools. Research conducted at the borderline of chemistry, biology and medicine indicates an urgent need for the synthesis of natural and non-natural saccharides and glycoconjugates of well-defined structure and composition. Due to their participation in many important biochemical processes, the increasing interest in them is justified. These compounds can serve as probes in research aimed at elucidating complex functions that carbohydrates play at the molecular level in living organisms, and additionally, can be used in the synthesis of new drugs based on carbohydrates [4,5,6,7,8,9,10,11,12,13,14]. An extremely important element of such building blocks is the glycosidic bond. *O*- and *N*-glycosidic bonds occur in nature, however, studies confirm their insufficient resistance to chemical and enzymatic hydrolysis. Replacing these bonds with C-C bonds has proved to be a very good solution. This modification increases the resistance required in therapeutic conditions while maintaining biological activity and good tolerance by living organisms [6,15,16,17,18,19,20,21,22,23,24,25,26,27,28,29,30,31,32]. An interesting and original idea is the coupling of sugar and aziridine, which leads to a completely new group of *C*-glycosides with promising biological properties. Chiral aziridines are useful intermediates in the synthesis of biologically significant compounds due to their ability to undergo nucleophilic ring opening reactions [33,34,35,36,37,38,39,40,41,42,43,44,45,46,47,48,49,50]. Considering our previous results on carbohydrate chemistry [51,52,53,54,55,56,57,58] and based on our experience in synthesis and catalytic activity in the asymmetric synthesis of chiral aziridines [59,60,61,62,63,64,65], we decided to couple aziridines to glycals with D-gluco and D-galacto configurations via *C*-glycosidic bonding, with the final formation of *C*-glycosyl-aminoethyl sulfide derivatives.

## 2. Results and Discussion

**Figure 1:**
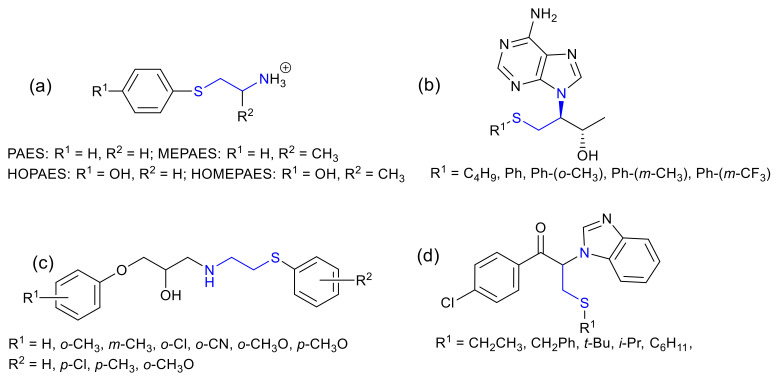
Literature examples of aminoethyl sulfides with biological properties. (**a**) PAES, or structurally similar derivatives; (**b**–**d**) compounds containing an aminoethyl sul-fide moiety.

Aminoethyl sulfide derivatives and, in particular, phenylaminoethylsulfides (PAES) have numerous applications due to their interesting biological properties. Derivatives of this type are synthetic substrates for dopamine β-hydroxylase (DBH; EC 1.14.17.1) [66,67]. As shown by May, PAES has indirect sympathomimetic activity in vivo and inhibits reflex tachycardia induced by vasodilating antihypertensive drugs. PAES, or structurally similar derivatives, may therefore be useful in the control of hypertension (Figure 1a) [68,69]. In addition, compounds containing an aminoethyl sulfide moiety have the ability to inhibit adenosine deaminase (ADA) (Figure 1b) [70], show β-adrenoreceptor blocking properties (Figure 1c) [71], and are used as inhibitors of DNA methyltransferases (Figure 1d) [72].

**Scheme 1:**
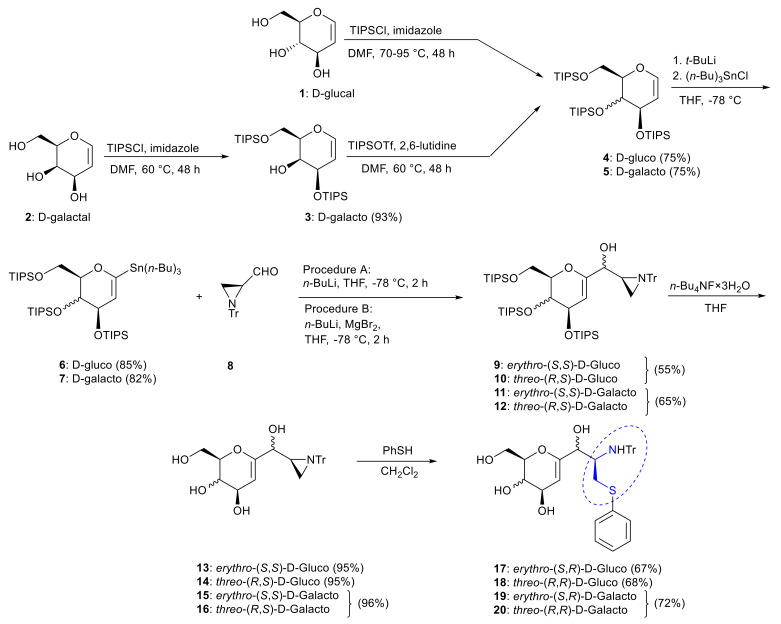
Synthesis of *C*-glycosyl-aminoethyl sulfide derivatives.

The new *C*-glycosyl-aminoethyl sulfide derivatives **17**–**20** with D-gluco and D-galacto configurations were easily prepared, according to Scheme 1.

Commercially available tri-*O*-acetyl-D-glucal and tri-*O*-acetyl-D-galactal were deacetylated in the presence of sodium methanolate in methanol and produced D-glucal (**1**) and D-galactal (**2**) in quantitative yields [73]. In the next step, saccharides **1** and **2** were treated with triisopropylsilyl chloride in the presence of imidazole in DMF to obtain the *O*-silylated derivatives **4** and **5 [74]**. However, under these conditions, the 4-hydroxyl group of the D-galacto derivative **2** remained unprotected. Protection of the free hydroxyl group required the use of triisopropylsilyl triflate and 2,6-lutidine (Scheme 1) [75]. Then, the protected derivatives **4** and **5** were subjected to 1-deprotonation with t*ert*-butyllithium and a reaction with tributyltin chloride [74]. The cases of 1-deprotonation of tribenzyl- and tris-(tertbutyldimethyl)-derivatives of D-glucal were also reported by several research groups, but they caused a number of problems and were characterized by low yields (10–30%) [76,77,78,79,80]. For this reason, tin derivatives **6** and **7** were prepared by deprotonation of the tris(triisopropylsilyl) glycals derivatives **4** and **5** with *tert*-butyllithium and quenching with tributyltin chloride (85% and 82% yields, respectively) [74]. A key step in the synthesis of *C*-glycosides was the coupling reaction of tin derivatives **6** and **7** with (*S*)-1-triphenylmethylaziridine-2-carbaldehyde (**8**), obtained according to literature procedures [81]. On the basis of the available databases, we can indicate only one example of the preparation of *C*-glycosides in the reaction of a tin derivative of D-glucal with an aldehyde obtained from diacetone-D-glucose. As reported by Whiting, the reaction resulted in isomeric *C*-disaccharides in a 2.1:1 ratio with a 31% yield from the stannane [74]. The reaction conducted under similar conditions, in the presence of *n*-BuLi in THF and at −78 °C for 2 h (Procedure A, Scheme 1) gave the desired *C*-glycoside analogs **9**–**12** in satisfactory yields. It seemed interesting to investigate both the stereochemistry of the addition of the organometallic compound to the aldehyde group of optically pure aziridine **8**, as well as to determine the influence of the metal cation on the stereochemistry of the reaction. Therefore, subsequent experiments were carried out in the presence of magnesium cation (Procedure B, Scheme 1).

Selectivity, in the addition reaction of organometallic reagents to aziridine 2-carboxyaldehydes, was described ten years ago by Jackson and Borhan [82]. In such addition reactions, new stereogenic centers are formed, leading to the formation of *syn*- or *anti*-adducts depending on the kind of metal. Those that are strong coordinators favor *syn* selectivity, which can be rationalized by a chelatation-based transition state, whereas metals that coordinate poorly, or conditions that suppress chelatation, favor the *anti*-adducts predicted by the Felkin–Anh model.

Initially, we performed the addition of the tin derivative of D-glucal **6** to aldehyde **8** obtaining a mixture of diastereoisomeric *C*-glycosides in the ratio *erythro*-**9**:*threo*-**10** = 1:3 with a total yield of 40% (Table 1, entry 1).

The introduction of magnesium cation into the reaction medium (Table 1, entry 2) resulted in an increase of yield to 55% and a simultaneous decrease in stereoselectivity (*erythro*-**9**:*threo*-**10** = 4:5). Importantly, diastereoisomeric products **9** and **10** were successfully purified and separated by flash chromatography, and all subsequent modifications were carried out on pure stereoisomers. Another experiment was carried out with the tin derivative of D-galactal **7** (Table 1, entry 3). The carbon–carbon bond formation process under these conditions occurred with a slightly higher yield (45%) and excellent stereoselectivity, compared to the analogous D-glucal derivative reaction. The same reaction performed in the presence of a magnesium cation resulted in *C*-glycosides **11** and **12** in an identical *erythro*/*threo* ratio of 1:9 (Table 1, entry 4) but with a much higher yield (65% total yield).

Unfortunately, the separation of diastereoisomeric products **11** and **12** was unsuccessful, so subsequent transformations were performed on their mixture.

The above results indicate that chelation is a minor factor in stereoselectivity. For galactal derivatives, the reactions take place mainly according to the Felkin–Ahn model (1:9). However, for glucal, stronger coordination is manifested by an increase in the share of the chelating model, but it does not exceed 50%. At the present stage of research, we do not have adequate experimental material that would allow for any generalizations. 

**Figure 2:**
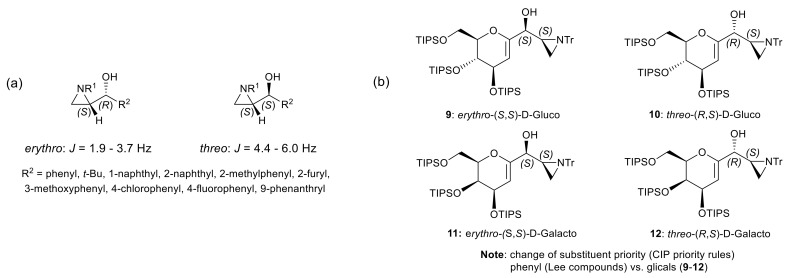
(**a**) Coupling constants of *erythro* and *threo* isomers according to literature data [83,84,85,86]; (**b**) configuration of *erythro* and *threo* obtained isomers **9**–**12**.

The absolute configurations of the resulting diastereoisomers **9**–**12** were determined by 1H NMR spectroscopy. According to literature data, the assignment of the absolute stereochemistry of the products is made by measuring the coupling constant between two neighboring methine protons, or the chemical shift of the methine proton on the hydroxyl-bearing carbon [83,84,85,86]. As reported by Lee [83], for the derivatives with the structure shown in the figure below (Figure 2), the coupling constant of the methine protons of the *threo* isomers (*anti* orientation of both protons) was always larger (*J* = 4.4–6.0 Hz) than those of *erythro* isomers (*J* = 1.9–3.7 Hz) in which protons are *syn* oriented. Moreover, the chemical shifts of the methine proton on the hydroxyl-bearing carbon of the *threo* isomers were always in higher field values (for example, 4.23 ppm for R^1^ = CH(Me)Ph) than those of the *erythro* isomers (4.66 ppm for R^1^ = CH(Me)Ph).

Based on the above reports, the absolute configuration of alcohols **9**–**12** was determined. Like the structures described by Lee, they possess the (*S*) configuration of the asymmetric carbon atom in the aziridine ring, which does not change during the reaction with the tin derivative of D-glucal **6** and D-galactal **7**. The recorded spectral data for D-glucal derivatives **9** and **10** indicate that diastereoisomer **9** of lower polarity (R_f_ = 0.45; petroleum ether:diethyl ether = 15:1) has the *erythro*-(*S*,*S*) configuration [4.40 (d, *J* = 2.2, C*H*OH)], while the second diastereoisomer **10** of higher polarity (R_f_ = 0.34; petroleum ether:diethyl ether = 15:1) is a *threo*-(*R*,*S*) isomer [3.92 ppm (d, *J* = 5.8, C*H*OH)] (Table 2). The stereochemistry of the products of the reaction of the tin derivative of D-galactal **7** with aldehyde **8** was determined in a similar manner. The methine proton signal at the carbon-containing hydroxyl group for the *threo* stereoisomer (*R*,*S*)-**12** occurred at lower ppm values (3.91 ppm) and the coupling constant of 5.8 Hz.

In contrast, the *erythro* isomer (*S*,*S*)-**11** gave a proton signal of the CHOH moiety, similar to the *erythro* isomer of glucal derivative **9**, at 4.33 ppm in the form of a doublet with the small coupling constant *J* = 2.4 Hz (Table 2).

In the next step, deprotection of hydroxyl groups was performed for pure diastereoisomers **9** and **10** with the D-gluko configuration and for the mixture of *erythro* and *threo* derivatives of D-galactal (**11**:**12** = 1:9). The reactions carried out in THF at room temperature in the presence of tetrabutylammonium fluoride [87] afforded *C*-glycosides **13**–**16** with free hydroxyl groups in the saccharide ring with yields of **13**:95%, **14**:95%, **15** and **16**:96%. The absolute configurations of the obtained *C*-glycosyl derivatives confirm the recorded spectral data. Although the coupling constants between two neighboring methine protons could not be determined (the signals of protons on the hydroxyl-bearing carbon appeared as broadened singlets), the chemical shift of the methine proton of C*H*OH moiety confirms the assigned configurations. The signals of the *threo* isomers **14** and **16** were in a higher field than those of the *erythro* isomers **13** and **15** (Table 2). The last step in the planned sequence of transformations (Scheme 1) was the reaction nucleophilic ring opening of aziridine with thiophenol. As is already known, the thiol group opens the aziridine ring particularly easily, and importantly, this reaction is fully regioselective, and the attack occurs on the less substituted carbon in aziridine [35,36]. All ring opening reactions were carried out in methylene chloride at room temperature, using three times the excess of thiophenol over the starting *C*-glycosides **13**–**16** (Scheme 1) [88,89]. Final products **17**–**20** were obtained with yields of 67% for **17**, 68% for **18**, 72% for **19** and **20**, respectively.

## 3. Materials and Methods

Commercially available chemicals used in this work were purchased from Sigma-Aldrich (Darmstadt, Germany) and were used as supplied, without additional purification. NMR spectra were recorded in CDCl_3_ on a Bruker Avance III (600 MHz for 1H NMR, 150 MHz for 13C NMR) (Billerica, MA, USA); coupling constants are reported in hertz (Hz). The rotations were measured using an Anton Paar MCP 500 polarimeter (Anton Paar GmbH, Graz, Austria). Melting points are uncorrected. Chromatographic purification of compounds was achieved with 230−400 mesh size silica gel. The progress of reactions was monitored by silica gel thin-layer chromatography plates (Merck TLC Silicagel 60 F254) (Merck Millipore, Darmstadt, Germany).

Copies of 1H and 13C NMR spectra of compounds **9**–**20** are included in the Supplementary Material.

### 3.1. General Procedure for the Synthesis of Glycals

A catalytic amount of MeONa (0.03 g, 0.57 mmol) was added to a solution of tri-***O***-acetyl-D-glycal (2.5 g, 9.18 mmol) in methanol (25 mL) and the resulting reaction mixture was stirred at rt. The progress of reactions was monitored by silica gel thin-layer chromatography plates. After 30 min, the solution was filtered on a Schott funnel over a resin layer (Amberlite^®^ IR120) and celite. Evaporation of the organic solvent afforded a pure product.

#### 3.1.1. D-Glucal (**1**)

Colorless solid, 1.67 g, 99% yield; R_f_ = 0.06 (hexane/ethyl acetate, 7:3); [α]_D_^20^ = −7.6 (c 0.9, CHCl_3_), {Lit. [90]: [α]_D_^20^ = −8.0 (c 1.19, H_2_O)}; m.p. = 54–56 °C, {Lit. 90]: m.p. = 58–60 °C}; δ_H_ (600 MHz, D_2_O): 3.61 (dd, 1H, *J* = 9.0, 7.1, H-4), 3.72–3.86 (m, 3H, H-5, 2H-6), 4.17 (dt, 1H, *J* = 7.1, 2.0, H-3), 4.73 (dd, 1H, *J* = 6.0, 2.0, H-2), 6.35 (dd, 1H, *J* = 6.0, 1.4, H-1).

1H NMR spectral data matched that reported by Crotti [91]. 

#### 3.1.2. D-Galactal (**2**)

Yellow oil, 1.70 g, 99% yield; R_f_ = 0.06 (hexane/ethyl acetate, 7:3); m.p. = 90–52 °C, {Lit. [92]: m.p. = 89–91 °C}; δ_H_ (600 MHz, CDCl_3_): 3.72 (d, 1H, *J* = 5.9, H-4), 3.77 (dd, 1H, *J* = 11.6, 5.2, H-6), 3.77–3.80 (m, 1H, H-5), 3.84 (dd, 1H, *J* = 11.6, 5.9, H-6), 3.90–3.95 (m, 3H, 3OH), 4.34–4.38 (m, 1H, H-3), 4.64 (dd, 1H, *J* = 6.2, 2.2, H-2), 6.36 (dd, 1H, *J* = 6.2, 1.6, H-1).

Spectroscopic data are in accordance with Refs. [75,93].

#### 3.1.3. 3,6-Di-*O*-(triisopropylsilyl)-D-galactal (**3**)

Colorless oil, 3.95 g, 93% yield; R_f_ = 0.79 (hexane/ethyl acetate, 20:1); [α]_D_^20^ = −33.81 (c 1.0, CHCl_3_), {Lit. [94]: [α]_D_^20^ = −34.0 (c 1.28, CHCl_3_)}; δ_H_ (600 MHz, CDCl_3_): 1.06–1.10 (m, 42H, TIPS-H), 1.55 (s, 1H, OH), 3.87 (dd, 1H, J = 7.2, 5.7, H-5), 3.92 (dd, 1H, *J* = 9.7, 5.9, H-6), 3.99–4.02 (m, 1H, H-4), 4.04 (dd, 1H, *J* = 9.7, 7.4, H-6), 4.56–4.61 (m, 2H, H-2, H-3), 6.34 (d, 1H, *J* = 4.9, H-1).

The synthesis and spectroscopic data are in accordance with Ref. [93].

#### 3.1.4. 3,4,6-Tris-*O*-(triisopropylsilyl)-D-glucal (**4**)

Colorless oil, 6.05 g, 75% yield; R_f_ = 0.95 (hexane/ethyl acetate, 25:1); [α]_D_^20^ = −17.77 (c 0.6, CHCl_3_), {Lit. [95]: [α]_D_^20^ = −21.4 (c 1.0, CHCl_3_)}; IR (film): 3066 (ν_=C-H_), 2942, 2867 (ν_C-H_), 1645 (ν_C=C_), 1068 (ν_C-O_); δ_H_ (600 MHz, CDCl_3_): 1.06 (s, 63H, TIPS-H), 3.82 (dd, 1H, *J* = 11.3, 3.8, H-6), 3.95 (dt, 1H, *J* = 5.2, 1.9, H-3), 4.04–4.08 (m, 2H, H-4, H-6), 4.22–4.25 (m, 1H, H-5), 4.80 (ddd, 1H, *J* = 6.6, 5.3, 1.7, H-2), 6.35 (d, 1H, *J* = 6.4, H-1); δ_C_ (150 MHz, CDCl_3_): 12.2, 12.5, 12.7 (CH), 18.1, 18.2, 18.3 (CH_3_), 62.3 (C-6), 65.3 (C-3), 70.5 (C-4), 80.9 (C-5), 100.5 (C-2), 143.1 (C-1).

The synthesis and spectroscopic data are in accordance with Refs. [74,95].

#### 3.1.5. 3,4,6-Tri-*O*-(triisopropylsilyl)-D-galactal (**5**)

Colorless oil, 1.5 g, 75% yield; R_f_ = 0.95 (hexane/ethyl acetate, 20:1); [α]_D_^20^ = −17.07 (c 0.6, CHCl_3_); IR (film): 3064, 3018 (ν_=C-H_), 2944, 2867 (ν_C-H_), 1641 (ν_C=C_), 1087 (ν_C-O_); δ_H_ (600 MHz, CDCl_3_): 1.04–1.17 (m, 63H, TIPS-H), 4.02–4.40 (m, 5H, H-3, H-4, H-5, 2H-6), 4.80 (bs, 1H, H-2), 6.24 (d, 1H, *J* = 6.1, H-1); δC (150 MHz, CDCl_3_): 11.9, 12.0, 12.2 (CH), 18.0, 18.1, 18.2, 18.4 (CH_3_), 62.8 (C-6), 68.1 (C-3), 74.2 (C-4), 86.2 (C-5), 98.1 (C-2), 142.9 (C-1). 

The synthesis and spectroscopic data are in accordance with Refs. [93,95].

### 3.2. General Procedure for the Synthesis of Tributhyltin Derivatives of Glycals

The tributhyltin derivatives were synthesized according to a literature procedure [11]. Where 3,4,6-Tris-*O*-(triisopropylsilyl)-D-glycal **4** or **5** (1.0 g, 1.62 mmol) was dissolved in dry THF (4 mL) and the solution was stirred under nitrogen, cooled to −78 °C, and treated with *t*-BuLi (1.7 mol·dm^−3^, 3.82 mL, 6.5 mmol) in one addition. The solution was warmed to 0 °C and stirred for 1.5 h, then was cooled to −78 °C, and tributyltin chloride (1.1 mL, 4.1 mmol) was added. The solution was again warmed to 0 °C and stirred for 45 min before the reaction was quenched with water (10 mL). The solution was poured into diethyl ether (10 mL) and the organic phase was separated. The aqueous phase was extracted with diethyl ether and the combined extracts were washed successively with water (10 mL) and brine (10 mL), dried over anhydrous MgSO_4_ and evaporated. The crude product was purified by column chromatography (hexane/ethyl acetate, 25:1).

#### 3.2.1. 1-(Tributylstannyl)-3,4,6-tris-*O*-(triisopropylsilyl)-D-glucal (**6**)

Colorless oil, 0.98 g, 85% yield, R_f_ = 0.94 (hexane/ethyl acetate, 25:1); [α]_D_^20^ = −23.87 (c 0.5, CHCl_3_); IR (film): 2942, 2867 (ν_C-H_), 1654 (ν_C=C_), 1068 (ν_C-O_); δ_H_ (600 MHz, CDCl_3_): 0.86–0.94 (m, 15H, *n*-Bu), 1.06 (s, 63H, TIPS-H), 1.27–1.34 (m, 6H, *n*-Bu), 1.48–1.55 (m, 6H, *n*-Bu), 3.85 (dt, 1H, *J* = 5.0, 2.6, H-3), 3.91 (dd, 1H, *J* = 11.3, 5.0, H-6), 3.96 (dd, 1H, *J* = 11.3, 7.0, H-6), 4.06–4.08 (m, 1H, H-4), 4.08–4.12 (m, 1H, H-5), 4.83 (dd, 1H, *J* = 5.2, 1.6, H-2); δ_C_ (150 MHz, CDCl_3_): 9.7 (*C*H_2_CH_2_CH_2_CH_3_), 12.3, 12.7, 12.8 (*C*H(CH_3_)_2_), 13.8 (*C*H_3_CH_2_CH_2_CH_2_), 18.2, 18.3, 18.4 (CH(*C*H_3_)_2_), 27.5 (CH_3_*C*H_2_CH_2_CH_2_), 29.1 (CH_3_CH_2_*C*H_2_CH_2_), 62.6 (C-6), 65.3 (C-3), 70.5 (C-4), 80.8 (C-5), 111.5 (C-2), 162.6 (C-1).

1H and 13C NMR spectral data matched that reported by Whiting [74].

#### 3.2.2. 1-(Tributylstannyl)-3,4,6-tris-*O*-(triisopropylsilyl)-D-galactal (**7**)

Colorless oil, 0.95 g, 82% yield; R_f_ = 0.95 (hexane/ethyl acetate, 25:1); [α]_D_^20^ = −27.82 (c 0.5, CHCl_3_); IR (film): 2942, 2867 (ν_C-H_), 1641 (ν_C=C_), 1072 (ν_C-O_); δ_H_ (600 MHz, CDCl_3_): 0.89–0.93 (m, 15H, *n*-Bu), 1.05–1.13 (m, 63H, TIPS-H), 1.30–1.36 (m, 6H, *n*-Bu), 1.50–1.56 (m, 6H, *n*-Bu), 4.06–4.26 (m, 5H, H-3, H-4, H-5, 2H-6), 4.84 (bs, 1H, H-2); δ_C_ (150 MHz, CDCl_3_): 9.7 (*C*H_2_CH_2_CH_2_CH_3_), 12.2, 12.8 (*C*H(CH_3_)_2_), 13.8 (*C*H_3_CH_2_CH_2_CH_2_), 18.2, 18.5 (CH(*C*H_3_)_2_), 27.5 (CH_3_*C*H_2_CH_2_CH_2_), 29.1 (CH_3_CH_2_*C*H_2_CH_2_), 61.4 (C-6), 64.7 (C-3), 70.6 (C-4), 81.1 (C-5), 112.9 (C-2), 162.6 (C-1); elementar analysis: C_45_H_96_O_4_Si_3_Sn (904.56 g/mol) calculated: C% 59.77, H% 10.70; found: C% 59.94, H% 10.86.

### 3.3. General Procedure for the Reaction of Derivatives of Glycals with Aziridine Aldehyde ***8***

Procedure A: 0.25 g (0.28 mmol) of tributhyltin derivative **6** or **7** was dissolved in 1.3 mL of dry THF and the solution was stirred under argon, cooled to −78 °C, and treated with *n*-BuLi (0.25 mL, 0.33 mmol) added dropwise. The solution was stirred at this temperature for 15 min, then 0.10 g (0.33 mmol) of aldehyde **8,** previously dissolved in 1 mL of dry THF, was added, and stirred for 1.5 h before the reaction was quenched with 5 mL of water. The solution was poured into methylene chloride (10 mL), the organic phase was separated and washed successively with water (3 × 5 mL) and brine (5 mL) and finally dried over anhydrous MgSO_4_, filtered and evaporated. The crude product was purified by column chromatography.

Procedure B: Preparing of MgBr_2_: 0.049 g (12 mmol) of magnesium turnings was placed in 6 mL of dry THF under an argon atmosphere, and then 0.17 mL (12 mmol) of 1,2-dibromoethane was added. The reaction was gently heated until the magnesium was completely dissolved. Then, 0.25 g (0.28 mmol) of tributhyltin derivative **6** or **7** was dissolved in 1.3 mL of dry THF and the solution was stirred under argon, cooled to −78 °C, and treated with *n*-BuLi (0.25 mL, 0.33 mmol) added dropwise. The solution was stirred at this temperature for 15 min, then 1 mL (0.33 mmol) previously prepared MgBr_2_ solution was added, continuing stirring for the next 15 min. In the next step, 0.10 g (0.33 mmol) of aldehyde **8** dissolved in 1 mL of dry THF was added and stirred for 1.5 h before the reaction was quenched with 5 mL of water. The solution was poured into methylene chloride (10 mL), the organic phase was separated and washed successively with water (3 × 5 mL) and brine (5 mL) and finally dried over anhydrous MgSO_4_, filtered and evaporated. The crude product was purified by column chromatography.

The reaction of the tin derivative of D-glucal **4** and D-galactal **5** with the aldehyde **8** resulted in a mixture of *erythro*-(*S*,*S*) and *threo*-(*R*,*S*) diastereoisomers. Pure stereoisomers of D-glucal **9** and **10** were isolated by flash chromatography on the apparatus Reveleris ^®^X2.

#### 3.3.1. *erythro*-(*S*)-[3,4,6-Tris-*O*-(triisopropylsilyl)-D-glucal-1-yl][(*S*)-1-triphenylmethylaziridin-2-yl]methanol (**9**)

Colorless solid, 55% yield; R_f_ = 0.45 (petroleum ether/diethyl ether, 15:1); [α]_D_^20^ = −13.04 (c 0.5, CHCl_3_) IR (KBr): 3479 (ν_O-H_), 3058, 3018 (ν_=C-H_), 2925, 2865 (ν_C-H_), 1596, 1469 (ν_CAr-CAr_), 1099 (ν_C-O_); δH (600 MHz, CDCl_3_): 0.87–0.93 (m, 21H, TIPS-H), 0.97 (d, 1H, *J* = 6.4, CH_2_N), 0.98–1.07 (m, 42H, TIPS-H), 1.84 (d, 1H, *J* = 3.1, CH_2_N), 1.92 (ddd, 1H, *J* = 6.4, 3.1, 2.2, CHN), 3.50 (s, 1H, OH), 3.81 (dd, 1H, *J* = 11.2, 4.1, H-6), 3.92–3.95 (m, 1H, H-3), 3.96 (dd, 1H, J = 11.2, 7.6, H-6), 3.98–4.01 (m, 1H, H-4), 4.12–4.16 (m, 1H, H-5), 4.40 (d, 1H, *J* = 2.2, CHOH), 4.96 (d, 1H, *J* = 5.3, H-2), 7.18–7.23 (m, 3H, C_6_H_5_), 7.23–7.30 (m, 6H, C_6_H_5_), 7.40 (d, 6H, *J* = 7.6, C_6_H_5_); δ_C_ (150MHz, CDCl_3_): 12.2, 12.5, 12.5 (TIPS-C), 18.1, 18.2, 18.3, 18.4 (TIPS-C), 22.5 (CH_2_N), 34.8 (CHN), 62.3 (C-6), 65.9 (C-3), 66.6 (CHOH), 70.2 (C-4), 74.1 (*C*(C_6_H_5_)_3_), 81.2 (C-5), 94.5 (C-2), 127.0, 127.8, 129.4 (C_6_H_5_), 144.3 (C_6_H_5_), 152.5 (C-1); HRMS (EI): calculated for C_55_H_89_NO_5_Si_3_ M^.+^ 928.6127; found 928.6112.

#### 3.3.2. *threo*-(*R*)-[3,4,6-Tris-O-(triisopropylsilyl)-D-glucal-1-yl][(*S*)-1-triphenylmethylaziridin-2-yl]methanol (**10**)

Colorless solid, 55% yield; R_f_ = 0.34 (petroleum ether/diethyl ether, 15:1); [α]_D_^20^ = −17.29 (c 0.6, CHCl_3_); IR (KBr): 3457 (ν_O-H_), 3058, 3020 (ν_=C-H_), 2943, 2866 (ν_C-H_), 1675 (ν_C=C_), 1520,1464 (ν_CAr-CAr_), 1062 (ν_C-O_); δ_H_ (600 MHz, CDCl_3_): 0.95–1.04 (m, 63H, TIPS-H), 1.10 (d, 1H, *J* = 6.4, CH_2_N), 1.58 (ddd, 1H, *J* = 6.4, 5.8, 3.0, CHN), 1.83 (d, 1H, *J* = 3.0, CH_2_N), 2.41 (s, 1H, OH), 3.80 (d, 2H, *J* = 6.2, 2H-6), 3.92 (d, 1H, *J* = 5.8, CHOH), 3.95–3.98 (m, 1H, H-3), 4.01–4.04 (m, 1H, H-4), 4.15–4.19 (m, 1H, H-5), 4.88 (d, 1H, *J* = 4.4, H-2), 7.16–7.20 (m, 3H, C_6_H_5_), 7.22–7.27 (m, 6H, C_6_H_5_), 7.50 (d, 6H, *J* = 7.6, C_6_H_5_); δ_C_ (150 MHz, CDCl_3_): 12.1, 12.5, 12.6 (TIPS-C), 18.1, 18.2, 18.3, 18.4 (TIPS-C), 25.2 (CH_2_N), 37.3 (CHN), 61.7 (C-6), 66.2 (C-3), 70.2 (C-4), 73.9 (*C*(C_6_H_5_)_3_), 75.0 (CHOH), 81.0 (C-5), 96.6 (C-2), 126.8, 127.6, 129.7 (C_6_H_5_), 144.6 (C_6_H_5_), 151.9 (C-1); HRMS (EI): calculated for C_55_H_89_NO_5_Si_3_ M^+^ 928.6127; found 928.6112.

#### 3.3.3. *erythro*-(*S*)-[3,4,6-Tris-*O*-(triisopropylsilyl)-D-galactal-1-yl][(*S*)-1-triphenylmethylaziridin-2-yl]methanol (**11**) and *threo*-(*R*)-[3,4,6-Tris-*O*-(triisopropylsilyl)-D-galactal-1-yl][(*S*)1-triphenylmethylaziridin-2-yl]methanol (**12**)

Colorless solid, 65% yield; R_f_ = 0.47 (hexane/ethyl acetate, 25:1); [α]_D_^20^ = −22.42 (c 0.3, CHCl_3_); IR (KBr): 3457 (ν_O-H_), 3058, 3018 (ν_=C-H_), 2927, 2865 (ν_C-H_), 1672 (ν_C=C_), 1596, 1436 (ν_CAr-CAr_), 1097 (ν_C-O_); δ_H_ (600 MHz, CDCl_3_): 0.91–1.09 (m, 63H, TIPS-H), 1.15 (d, 1H, *J* = 6.4, CH_2_N), 1.47 (ddd, 1H, *J* = 6.4, 5.8, 2.9, CHN), 1.97 (bs, 1H, CH_2_N), 2.02 (d, 1H, *J* = 3.1, CH_2_N, *erythro*), 2.38 (s, 1H, OH), 3.58 (s, 1H, OH, *erythro*), 3.91 (d, 1H, *J* = 4.8, CHOH), 3.94–4.30 (m, 5H, H-3, H-4, H-5, 2H-6), 4.33 (d, 1H, *J* = 2.4, CHOH*, eryhtro*), 4.79 (bs, 1H, H-2), 4.90 (bs, 1H, H-2, *erythro*), 7.17–7.22 (m, 3H, C_6_H_5_), 7.23–7.30 (m, 6H, C_6_H_5_), 7.41 (d, *J* = 7.7, C_6_H_5_, *erythro*) 7.50 (d, 6H, *J* = 7.8, C_6_H_5_); δ_C_ (150 MHz, CDCl_3_): 12.1, 12.7 (TIPS-C), 18.1, 18.2, 18.3, 18.4, 18.4 (TIPS-C), 29.9 (CH_2_N), 37.4 (CHN), 61.0 (C-6), 64.3 (C-3), 70.2 (C-4), 73.6 (*C*(C_6_H_5_)_3_), 73.8 (CHOH), 80.8 (C-5), 97.6 (C-2), 99.1 (C-2, *erythro*), 126.9, 127.0, 127.7, 127.8, 128.9, 129.4, 129.6, 131.0 (C_6_H_5_), 144.3 (C_6_H_5_, *erythro*), 144.5 (C6H5), 152.7 (C-1); elementar analysis: C_55_H_89_NO_5_Si_3_ (928.56 g/mol) calculated: C% 71.14, H% 9.66, N% 1.51; found: C% 71.08, H% 9.66, N% 1.48.

### 3.4. General Procedure for Deprotection of Hydroxyl Groups

In the round bottom flask, there are 4 equivalents of tetrabutylammonium fluoride in 2.5 mL of dry THF, then the flask was secured with a septum and CaCl_2_ tube. 1 Equivalent of the compound **9**–**12** was dissolved in 2.5 mL of dry THF and added slowly to the tetrabutylammonium fluoride solution. Stirring was continued for 18 h at room temperature. After this time, the solvent was evaporated, and the residue was dissolved in ethyl acetate (15 mL), washed with brine (15 mL) and then dried over anhydrous MgSO_4_. After filtration and evaporation of the solvent, the product was purified by column chromatography using ethyl acetate and methanol (25:1).

#### 3.4.1. *erythro*-(*S*)-[D-Glucal-1-yl][(*S*)-1-triphenylmethylaziridin-2-yl]methanol (**13**)

Colorless solid, 95% yield; R_f_ = 0.56 (ethyl acetate/methanol, 25:1); [α]_D_^20^ = −10.13 (c 0.6, CHCl_3_); IR (KBr): 3450 (ν_O-H_), 3052 (ν_=C-H_), 2970, 2855 (ν_C-H_), 1627 (ν_C=C_), 1592, 1466 (ν_CAr-CAr_), 1067 (ν_C-O_); δ_H_ (600 MHz, CDCl_3_): 1.12 (d, 1H, *J* = 5.2, CH_2_N), 1.69 (m, 1H, CHN), 1.84 (d, 1H, *J* = 2.4, CH_2_N), 3.65–3.71 (m, 2H, H-4, H-5), 3.77 (d, 1H, *J* = 11.7, H-6), 3.82 (d, 1H, *J* = 11.7, H-6), 4.08–4.17 (m, 1H, H-3), 4.30 (d, 1H, *J* = 3.1, CHOH), 4.74 (bs, 1H, H-2), 7.19 (t, 3H, *J* = 7.2, C_6_H_5_), 7.22–7.27 (m, 6H, C_6_H_5_), 7.40 (d, 6H, *J* = 7.7, C_6_H_5_); δ_C_ (150 MHz, CDCl_3_): 29.8 (CHN), 34.4 (CHN), 61.2 (C-6), 67.9 (CHOH), 69.4 (C-4), 70.0 (C-3), 74.1 (*C*(C_6_H_5_)_3_), 78.5 (C-5), 99.6 (C-2), 127.1, 127.8, 129.4 (C_6_H_5_), 144.1 (C_6_H_5_), 154.3 (C-1); MS-EI m/z: 482.1 [M + Na]^+^; TOF MS ES^+^ calculated for C_28_H_29_NO_5_Na [M]^.+^ 482.1943; found 482.1950.

#### 3.4.2. *threo*-(*R*)-[D-Glucal-1-yl][(*S*)-1-triphenylmethylaziridin-2-yl]methanol (**14**)

Colorless solid, 95% yield; R_f_ = 0.53 (ethyl acetate/methanol, 25:1); [α]_D_^20^ = −7.62 (c 0.4, CHCl_3_); IR (KBr): 3453 (ν_O-H_), 3048 (ν_=C-H_), 2955, 2868 (ν_C-H_), 1634 (ν_C=C_), 1575, 1472 (ν_CAr-CAr_), 1059 (ν_C-O_); δ_H_ (600 MHz, CDCl_3_): 1.12 (d, 1H, *J* = 5.3, CH_2_N), 1.62 (m, 1H, CHN), 1.79 (d, 1H, *J* = 2.3, CH_2_N), 3.48 (dd, 1H, *J* = 8.7, 7.2, H-4), 3.58 (d, 1H, *J* = 11.6, H-6), 3.63 (d, 4H, *J* = 9.6, H-5), 3.74 (d, 1H, *J* = 11.6, H-6), 3.94 (bs, 1H, CHOH), 4.11 (d, 1H, *J* = 7.2, H-3), 4.80 (bs, 1H, H-2), 7.13 (t, 3H, *J* = 7.1, C_6_H_5_), 7.17–7.23 (m, 6H, C_6_H_5_), 7.38 (d, 6H, *J* = 7.3, C_6_H_5_); δ_C_ (150 MHz, CDCl_3_): 32.1 (CHN), 35.8 (CHN), 60.5 (C-6), 70.0 (C-3, C-4), 70.7 (CHOH), 74.0 (*C*(C_6_H_5)3_), 78.2 (C-5), 99.9 (C-2), 127.0, 127.7, 128.3, 128.9, 129.7, (C_6_H_5_), 144.2 (C_6_H_5_), 154.8 (C-1); MS-EI m/z: 482.1 [M + Na]^+^; TOF MS ES+ calculated for C_28_H_29_NO_5_Na [M]^.+^ 482.1943; found 482.1942.

#### 3.4.3. *erythro*-(*S*)-[D-Glalactal-1-yl][(*S*)-1-triphenylmethylaziridin-2-yl]methanol (**15**) and *threo*-(*R*)-[D-Galactal-1-yl][(*S*)-1-triphenylmethylaziridin-2-yl]methanol (**16**)

Colorless solid, 96% yield, R_f_ = 0.54 (ethyl acetate/methanol, 25:1); [α]_D_^20^ = −3.85 (c 0.4, CHCl_3_); IR (KBr): 3477 (ν_O-H_), 3063 (ν_=C-H_), 2975 (ν_C-H_), 1653 (ν_C=C_), 1543, 1491 (ν_CAr-CAr_), 1068 (ν_C-O_); δ_H_ (600 MHz, CDCl_3_): 1.27 (d, 1H, *J* = 5.2, CH_2_N), 1.66 (bs, 1H, OH), 1.89–1.94 (m, 1H, CHN), 1.97 (d, 1H, *J* = 3.4, CHN), 2.20 (bs, 1H, OH), 2.37 (bs, 1H, OH), 3.38 (bs, 1H, OH), 3.77 (dd, 1H, *J* = 12.5, 5.7, H-6), 3.82–3.86 (m, 2H, H-4, H-5), 3.87 (dd, 1H, *J* = 12.5, 5.3, H-6), 4.04 (bs, 1H, CHOH), 4.28 (bs, 1H, CHOH, *erythro*), 4.29–4.34 (m, 1H, H-3), 4.79 (bs, 1H, *J* = 4.5, H-2, *erythro*), 4.89 (dd, 1H, *J* = 5.7, 1.6, H-2), 7.23 (t, 3H, *J* = 7.2, C_6_H_5_), 7.29 (t, 6H, *J* = 7.1, C_6_H_5_), 7.42 (d, *J* = 7.8, C_6_H_5_, *erythro*), 7.46 (d, 6H, *J* = 7.5, C*_6_*H_5_); δ_C_ (150 MHz, CDCl_3_): 29.5 (CHN, *erythro*), 29.8 (CHN), 34.8 (CHN, *erythro*), 35.4 (CHN), 62.8 (C-6, *erythro*), 62.9 (C-6), 64.6 (C-3, *erythro*), 64.7 (C-3), 66.4 (C-4), 67.7 (CHOH), 68.4 (CHOH, *erythro*), 73.9 (*C*(C_6_H_5_)_3_), 74.2 (*C*(C_6_H_5_)_3_, *erythro*), 76.6 (C-5, *erythro*), 76.7 (C-5), 97.6 (C-2), 99.2 (C-2, *erythro*), 127.2, 127.7, 129.8 (C_6_H_5_), 127.8, 129.5 (C_6_H_5_, *erythro*), 144.0 (C_6_H_5_), 144.1 (C_6_H_5_, *erythro*), 154.2 (C-1, *erythro*), 155.7 (C-1); MS-EI m/z: 482.1 [M + Na^]+^; TOF MS ES^+^ calculated for C_28_H_29_NO_5_Na [M]^.+^ 482.1943; found 482.1952.

### 3.5. General Procedure for Aziridine Ring Opening Reaction

In a round bottom flask, 1 equivalent of compound **13**–**16** was dissolved in 1 mL of methylene chloride and then 3 equivalents of thiophenol were added. The mixture was stirred at room temperature for 2–6 h (controlled by TLC tests). The crude product was dissolved in methylene chloride and purified on a preparative plate using ethyl acetate and methanol (25:1).

#### 3.5.1. *erytro*-(1*S*,2*R*)-1-[(1-Hydroxy-3-(phenylthio)-2-(triphenylmethylamino)propyl)]-D-glucal (**17**)

White solid, 67% yield; R_f_ = 0.58 (ethyl acetate/methanol, 25:1); [α]_D_^20^ = −7.68 (c 0.6, CHCl_3_); IR (KBr): 3385 (ν_O-H_), 3083, 3057, 3031 (ν_=C-H_), 2923, 2852 (ν_C-H_), 1636 (ν_C=C_), 1594, 1576, 1521, 1447 (ν_CAr-CAr_), 644 (ν_C-S_); δ_H_ (600 MHz, CDCl_3_): 2.06 (bs, 4H, 4OH), 2.86–2.97 (m, 3H, CH_2_S, CHN), 3.49–3.59 (m, 2H, H-4, H-5) 3.63–3.58 (m, 1H, H-3), 3.69 (d, 1H, *J* = 12.5, H-6), 3.77 (d, 1H, *J* = 12.5, H-6), 4.12 (d, 1H, *J* = 5.1, CHOH), 4.70 (bs, 1H, H-2), 7.08 (d, 2H, *J* = 7.1, C_6_H_5_), 7.11–7.23 (m, 13H, C_6_H_5_), 7.46 (d, 6H, *J* = 7.6, C_6_H_5_); δ_C_ (150 MHz, CDCl_3_): 36.0 (CH_2_S), 54.1 (CHN), 61.3 (C-6), 69.8 (CHOH), 70.0 (C-3), 70.6 (C-4), 71.1 (*C*(C_6_H_5_)_3_), 77.8 (C-5), 98.9 (C-2), 126.2, 126.5, 1279, 128.8, 128.9, 129.9, 136.4 (C_6_H_5_), 146.3 (C_6_H_5_), 154.4 (C-1); MS-EI m/z: 592.7 [M + Na]^+^; TOF MS ES^+^ calculated for C_34_H_35_NO_5_NaS [M]^.+^ 592.2134; found 592.2150.

#### 3.5.2. *threo*-(1*R*,2*R*)-1-[(1-Hydroxy-3-(phenylthio)-2-(triphenylmethylamino)propyl)]-D-glucal (**18**)

Colorless solid, 68% yield; R_f_ = 0.51 (ethyl acetate/methanol, 25:1); [α]_D_^20^ = + 13.8 (c 0.4, CHCl_3_); IR (KBr): 3382 (ν_O-H_), 3079, 3043, 3031 (ν_=C-H_), 2918, 2843 (ν_C-H_), 1642 (ν_C=C_), 1589, 1571, 1520, 1437 (ν_CAr-CAr_), 637 (ν_C-S_); δ_H_ (600 MHz, CDCl_3_): 1.66 (bs, 4H, OH), 2.12 (dd, 1H, *J* = 12.8, 6.2, CH_2_S), 2.65 (dd, 1H, *J* = 12.8, 1.7, CH_2_S), 3.21 (t, 1H, *J* = 5.9, CHN), 3.58–3.69 (m, 2H, H-5, H-6), 3.81 (t, 1H, *J* = 9.4, H-4), 3.86–3.93 (m, 2H, H-6, CHOH), 4.12 (d, 1H, *J* = 7.3, H-3), 4.65 (s, 1H, H-2), 7.07 (d, 2H, *J* = 8.4, C_6_H_5_), 7.15–7.28 (m, 13H, C_6_H_5_), 7.48 (d, 6H, *J* = 7.4, C_6_H_5_); δ_C_ (150 MHz, CDCl_3_): 36.5 (CH_2_S), 54.3 (CHN), 60.6 (C-6), 68.3 (C-4), 69.7 (C-3), 71.2 (*C*(C_6_H_5_)_3_), 73.0 (CHOH), 79.0 (C-5), 104.0 (C-2), 126.5, 126.9, 128.2, 129.0, 130.1, 136.7 (C_6_H_5_), 146.2 (C_6_H_5_), 151.9 (C-1); MS-EI m/z: 529.2 [M + Na]^+^; TOF MS ES+ calculated for C_34_H_35_NO_5_NaS [M]^.+^ 592.2134; found 592.2156.

#### 3.5.3. *erytro*-(1*S*,2*R*)-1-[(1-Hydroxy-3-(phenylthio)-2-(triphenylmethylamino)propyl)]-D-galactal (**19**) and threo-((1*R*,2*R*)-1-[(1-hydroxy-3-(phenylthio)-2-(triphenylmethylamino)propyl)]-D-galactal (**20**)

Colorless solid, 72% yield, R_f_ = 0.58 (ethyl acetate/methanol, 25:1); [α]_D_^20^ = −3.67 (c 0.6, CHCl_3_); IR (KBr): 3390 (ν_O-H_), 3093, 3066, 3042 (ν_=C-H_), 2939, 2866 (ν_C-H_), 1655 (ν_C=C_), 1592, 1569, 1526, 1437 (ν_CAr-CAr_), 651 (ν_C-S_); δ_H_ (600 MHz, CDCl_3_): 1.97 (bs, 1H, OH, *threo*), 2.74 (s, 3H, 3OH, *threo*), 2.90 (dd, 1H, *J* = 15.3, 10.3, CH_2_S, *threo),* 3.14–3.19 (m, 2H, CH_2_S, CHN, *threo*), 3.73 (dd, 1H, *J* = 13.4, 3.4, H-6, *threo*), 3.86 (dd, 1H, *J* = 13.4, 1.1, H-6, *threo*), 3.94–3.97 (m, 1H, H-5, *threo*), 4.02–4.05 (m, 1H, H-3, *threo*), 4.14 (t, 1H, *J* = 4.1, H-4, *threo*), 4.20 (bs, 1H, CHOH, *threo*), 4.87 (d, 1H, *J* = 4.7, H-2, *erythro*), 5.05 (dd, 1H, *J* = 4.6, 1.0, H-2, *threo*), 7.17 (t, 3H, *J* = 7.4, C_6_H_5_, *threo*), 7.19–7.27 (m, 17H, C_6_H_5_, *threo*); δ_C_ (150 MHz, CDCl_3_): 39.7 (CH_2_S), 52.1 (CHN), 61.0 (C-6), 61.5 (C-3), 67.1 (C-4), 70.9 (CHOH), 76.2 (C-5), 82.2 (*C*(C_6_H_5_)_3_), 98.0 (C-2), 127.0, 127.4, 129.0, 129.4, 130.3 (C_6_H_5_), 147.0 (C_6_H_5_), 155.0 (C-1); MS-EI m/z: 592.1 [M + Na]^+^; TOF MS ES^+^ calculated for C_34_H_35_NO_5_NaS [M]^+^ 592.2134; found 592.2141.

## 4. Conclusions

In conclusion, we have developed a simple and stereoselective methodology for the synthesis of *C*-glycosyl-aminoethyl sulfide derivatives of potential biological interest by a reaction of tributhyltin derivatives **6** and **7** of glycals with aziridinecarboaldehyde **8** and the regioselective ring opening of a chiral aziridine with thiophenol. The absolute configurations of the resulting diastereoisomers were determined via 1H NMR spectroscopy. The obtained results indicate that chelation is a less important factor influencing stereoselectivity. For galactal derivatives, the reactions proceed mainly according to the Felkin–Anh model, leading predominantly to the *threo* product. However, for glucal derivative, a higher contribution of “chelation-controlled” carbon–carbon bond formation was observed, which results in an increase of the *erythro* isomer. At the present stage of the study, we do not have adequate experimental material to explain the higher diastereoselectivity of the D-galacto derivative **7** in comparison to D-gluco **6**.

## Figures and Tables

**Table 1:** Reaction of tributhyltin derivatives of glycals **6** and **7** with aziridinecarboaldehyde **8,** according to Scheme 1.

Entry	Glycal	Procedure	Yield (%) ^1^	*erythro*:*threo*^2^
1	**6**	A	40	1:3
2	**6**	B	55	4:5
3	**7**	A	45	1:9
4	**7**	B	65	1:9

^1^ Isolated product. ^2^ Determined by 1H NMR analysis.

**Table 2:** Characteristic coupling constants and chemical shift values of diastereoisomers **9**–**16**.

Compound	C*H*OH δ (ppm)	C*H*N-C*H*OH *J* (Hz)	Compound	C*H*OH δ (ppm)
*erythro* (*S*,*S*)-**9**	4.40	2.2	(*S*,*S*)-**13**	4.30
*threo* (*R*,*S*)-**10**	3.92	5.8	(*R*,*S*)-**14**	3.94
*erythro* (*S*,*S*)-**11**	4.33	2.4	(*S*,*S*)-**15**	4.28
*threo* (*R*,*S*)-**12**	3.91	5.8	(*R*,*S*)-**16**	4.04

## Data Availability

Not applicable.

## Supplementary Materials

The following supporting information can be downloaded at: https://www.mdpi.com/article/10.3390/molecules27061764/s1, Figure S1: ^1^H NMR (600 MHz, CD_3_Cl) spectrum of **9**, Figure S2: ^13^C NMR (150 MHz, CD_3_Cl) spectrum of **9**, Figure S3: ^1^H-^1^H COSY spectrum of **9**, Figure S4: ^1^H-^13^C HMQC spectrum of **9**, Figure S5: ^1^H NMR (600 MHz, CD_3_Cl) spectrum of **10**, Figure S6: ^13^C NMR (150 MHz, CD_3_Cl) spectrum of **10**, Figure S7: ^1^H-^1^H COSY spectrum of **10**, Figure S8: ^1^H-^13^C HMQC spectrum of **10**, Figure S9: ^1^H NMR (600 MHz, CD_3_Cl) spectrum of **11** and **12**, Figure S10: ^13^C NMR (150 MHz, CD_3_Cl) spectrum of **11** and **12**, Figure S11: ^1^H NMR (600 MHz, CD_3_Cl) spectrum of **13**, Figure S12: ^13^C NMR (150 MHz, CD_3_Cl) spectrum of **13**, Figure S13: ^1^H NMR (600 MHz, CD_3_Cl) spectrum of **14**, Figure S14: ^13^C NMR (150 MHz, CD_3_Cl) spectrum of **14**, Figure S15: ^1^H NMR (600 MHz, CD_3_Cl) spectrum of **15** and **16**, Figure S16: ^13^C NMR (150 MHz, CD_3_Cl) spectrum of **15** and **16**, Figure S17: ^1^H NMR (600 MHz, CD_3_Cl) spectrum of **17**, Figure S18: ^13^C NMR (150 MHz, CD_3_Cl) spectrum of **17**, Figure S19: ^1^H NMR (600 MHz, CD_3_Cl) spectrum of **18**, Figure S20: ^13^C NMR (150 MHz, CD_3_Cl) spectrum of **18**, Figure S21: ^1^H NMR (600 MHz, CD_3_Cl) spectrum of **19** and **20**, Figure S22: ^13^C NMR (150 MHz, CD_3_Cl) spectrum of **19** and **20**.

## Sample Availability

Samples of the compounds are available from the authors.
