# Supplementary material for: Aziridine Ring Opening as Regio- and Stereoselective Access to C-Glycosyl-Aminoethyl Sulfide Derivatives
Source: Molecules. 2022 Mar 8;27(6):1764. doi: 10.3390/molecules27061764 (PMC8952378; doi:10.3390/molecules27061764)
Supplement: Supplementary file 1 [file molecules-27-01764-s001.zip › Supplementary Information.pdf]

Supplementary Information

Aziridine ring opening as regio- and stereoselective access to *C*-glycosyl-aminoethyl sulfide derivatives

Aleksandra Tracz, Martyna Malinowska, Stanisław Leśniak and Anna Zawisza*

Department of Organic and Applied Chemistry, University of Łódź, Tamka 12, 91-403 Łódź, Poland

*Corresponding author. Tel: (48) 42 6355802; Fax: (48) 42 6655162; E-mail address: azawisza@chemia.uni.lodz.pl

Table of contents

1. Copies of ^{1}H and ^{13}C NMR spectra of compounds **9-20**

1. Copies of ^{1}H and ^{13}C NMR spectra of compounds **9-20**

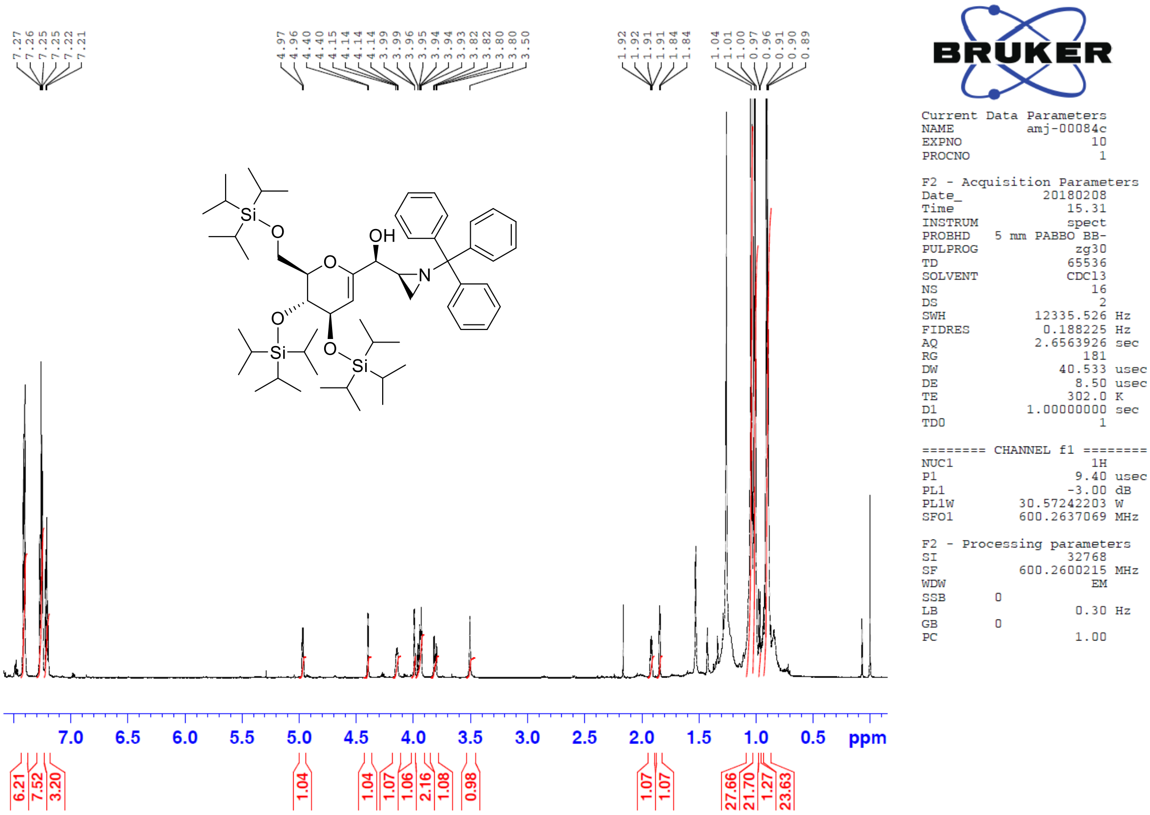

Figure S1. ^{1}H NMR (600 MHz, CD_3Cl) spectrum of **9**.

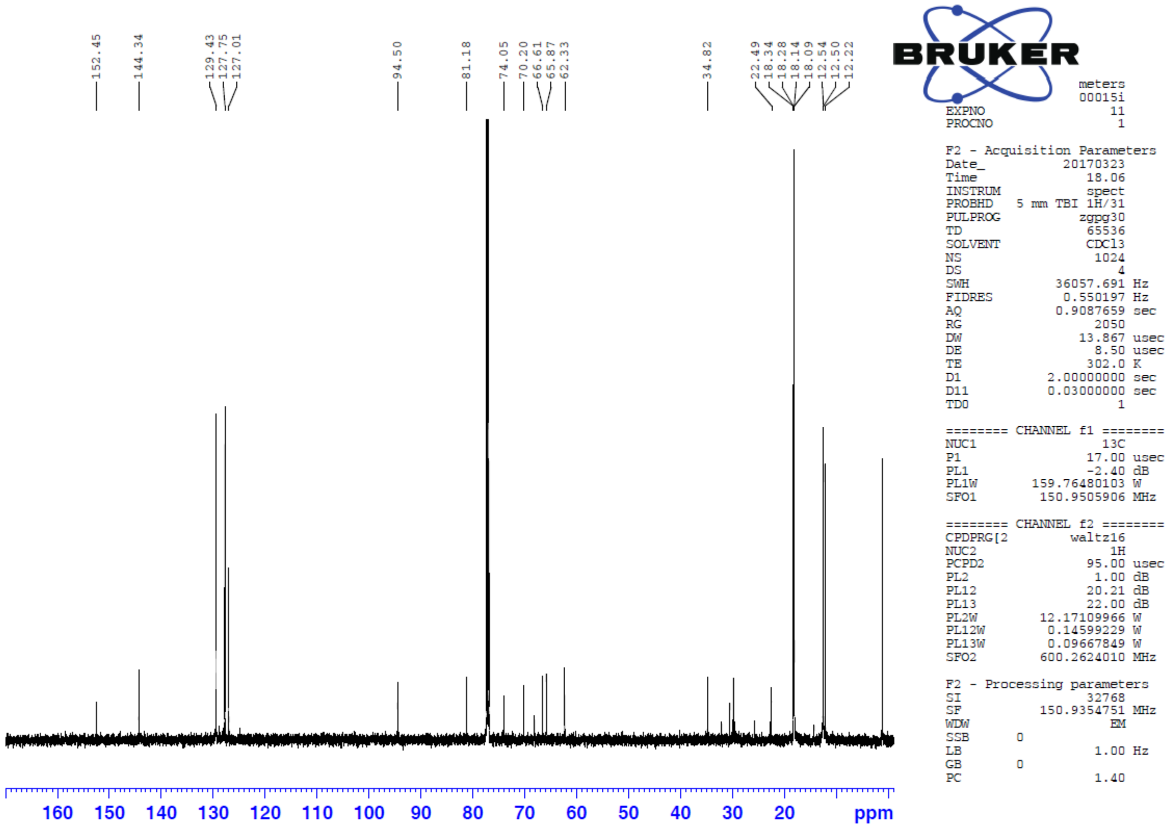

Figure S2. ^{13}C NMR (150 MHz, CD_3Cl) spectrum of **9**.

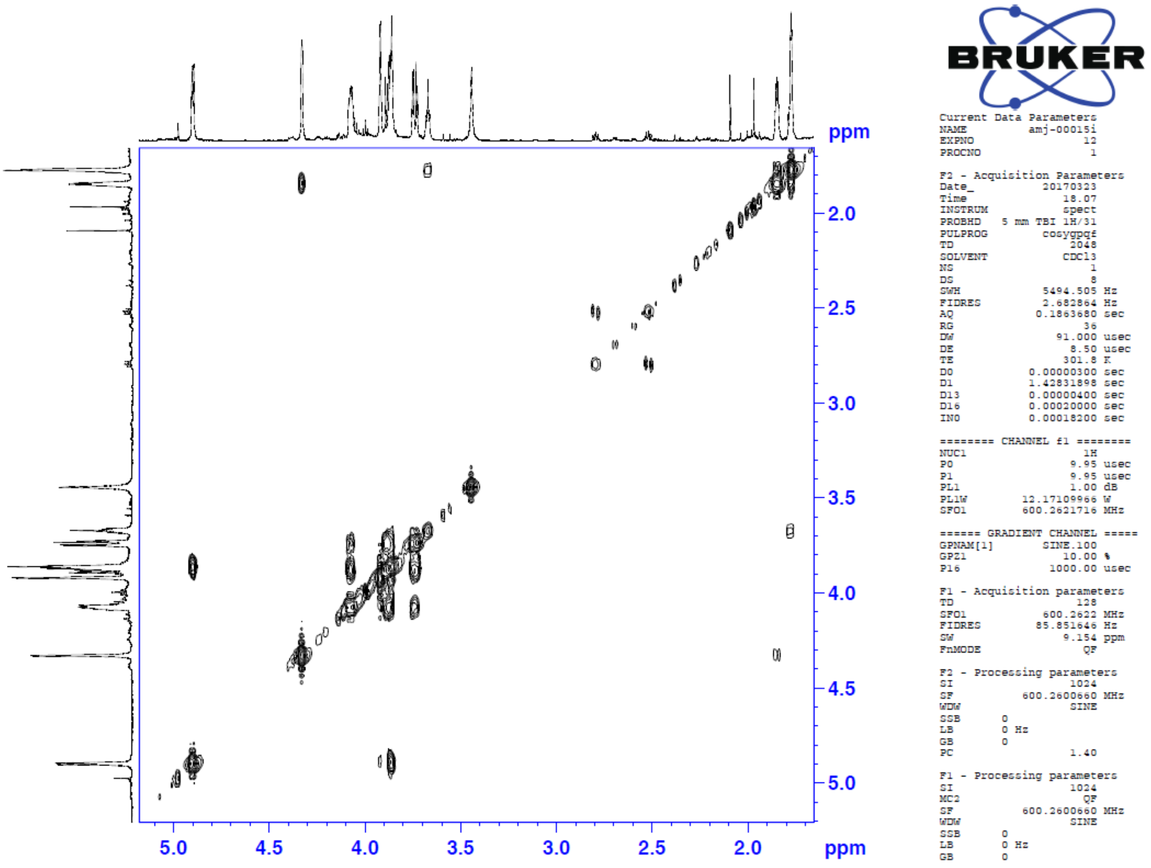

Figure S3. ^{1}H-^{1}H COSY spectrum of **9**.

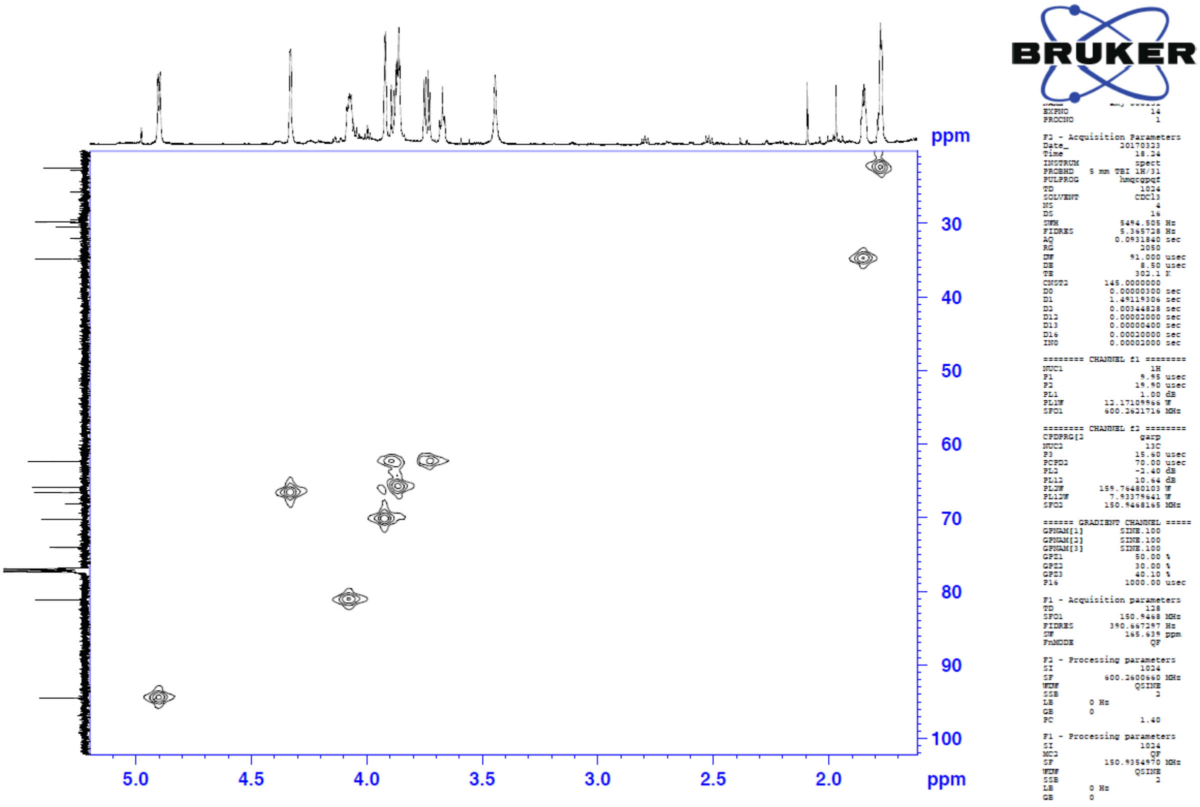

Figure S4. ^{1}H-^{13}C HMQC spectrum of **9**.

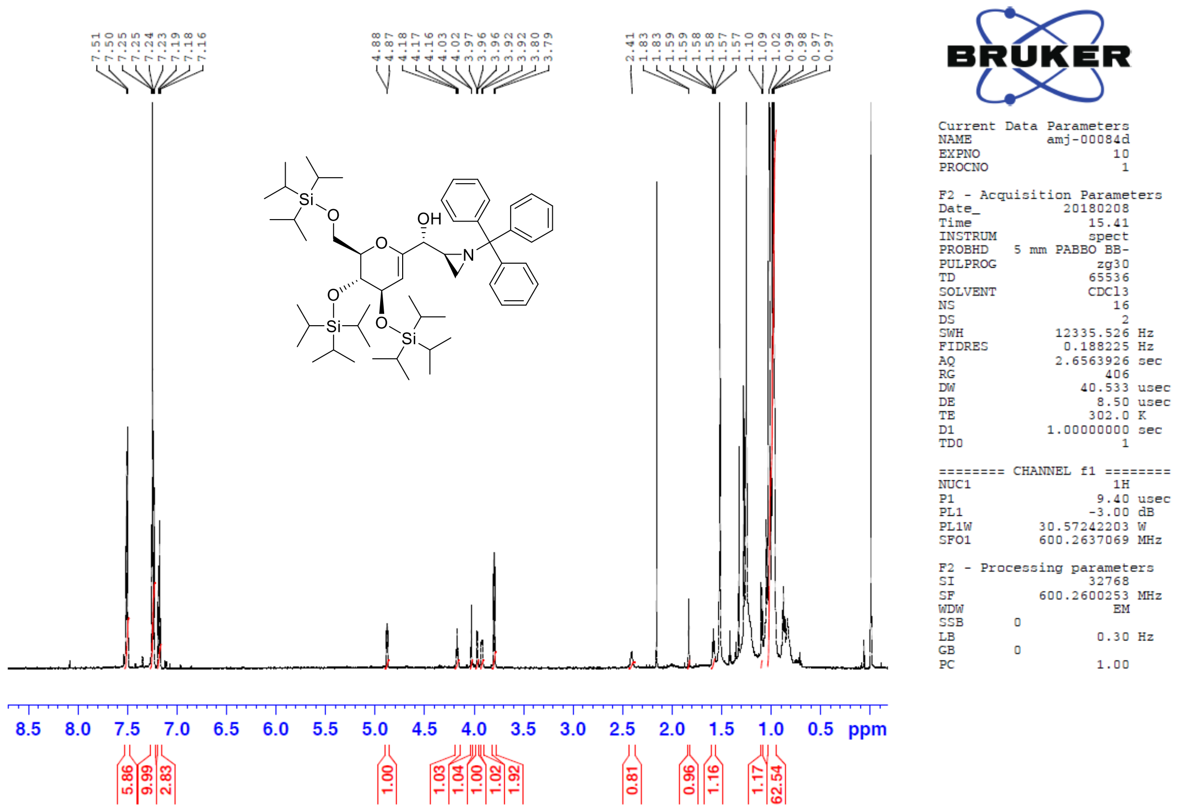

Figure S5. ^{1}H NMR (600 MHz, CD_3Cl) spectrum of **10**.

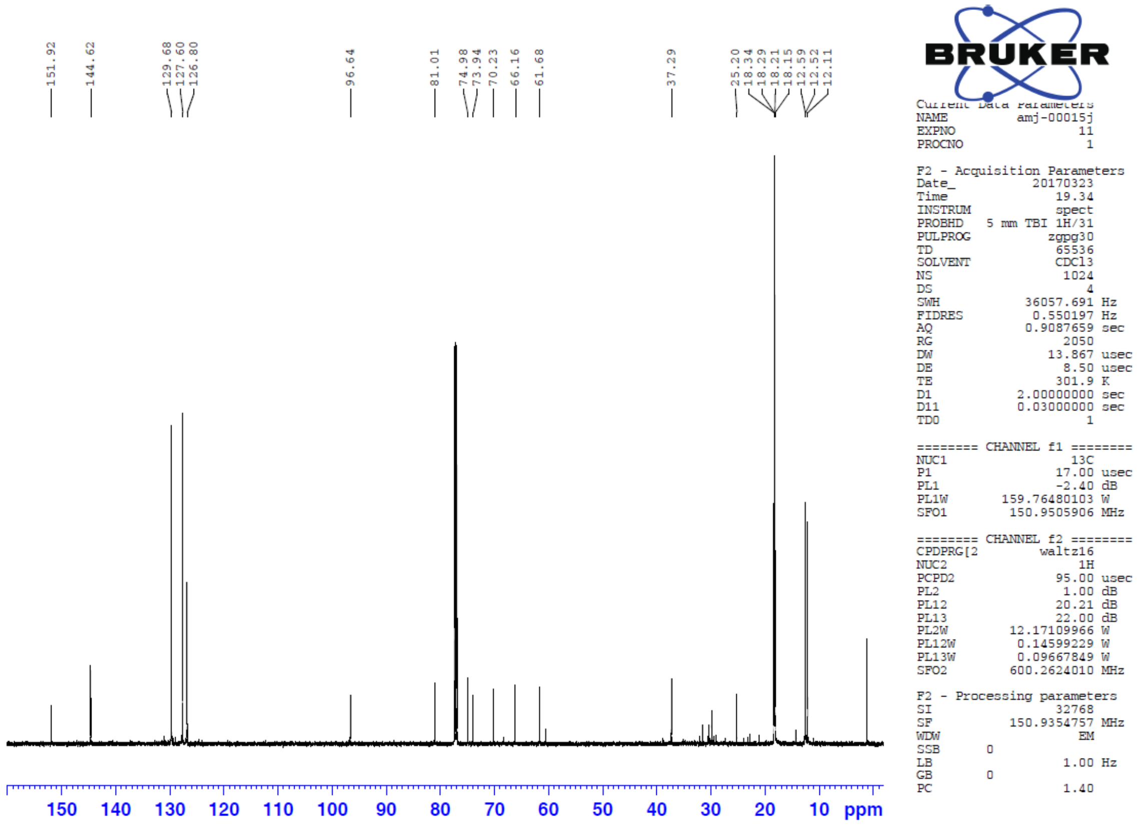

Figure S6. ^{13}C NMR (150 MHz, CD_3Cl) spectrum of **10**.

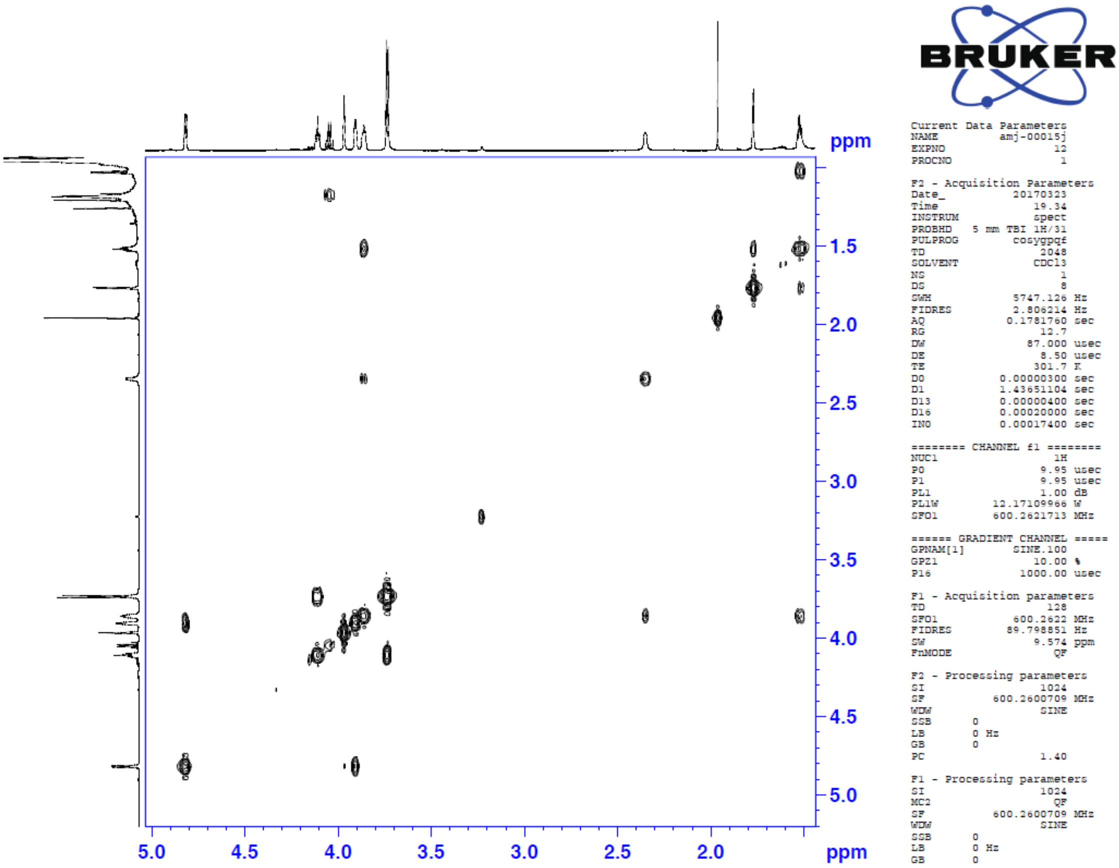

Figure S7. ^{1}H-^{1}H COSY spectrum of **10**.

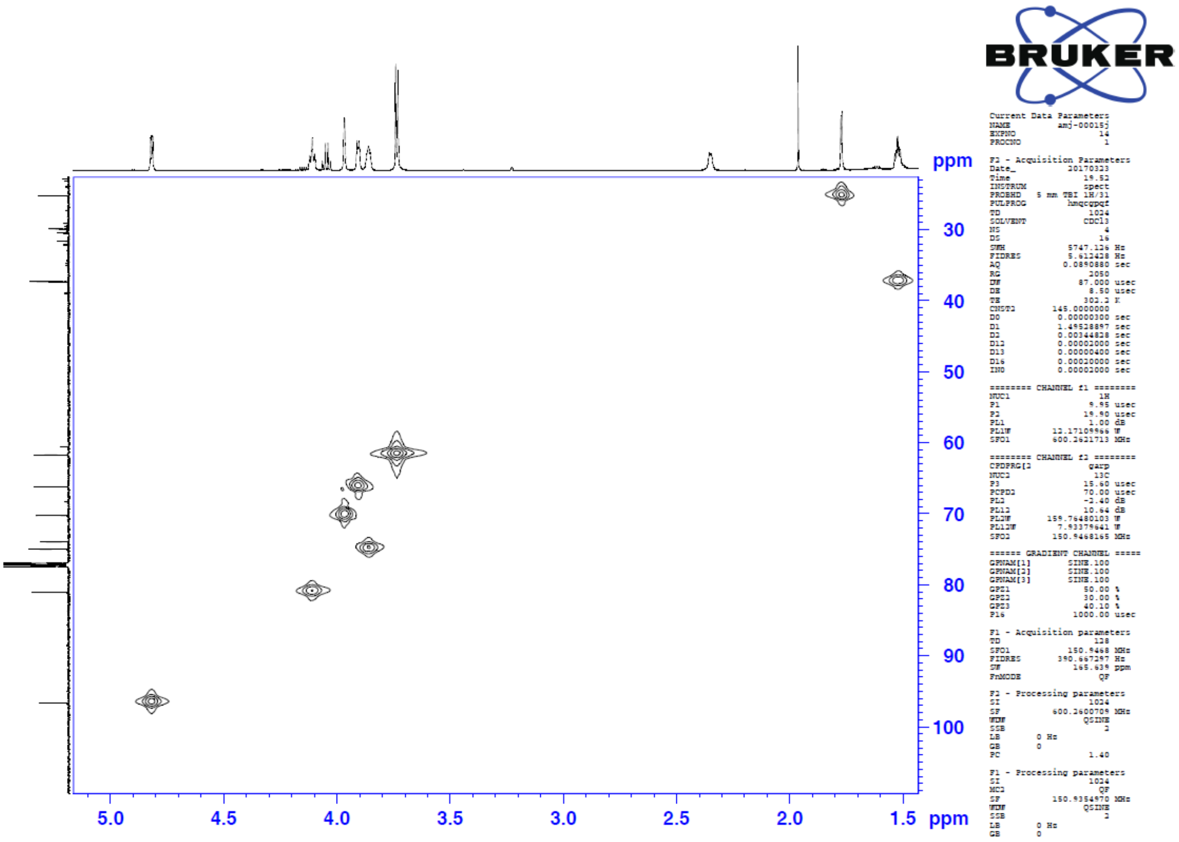

Figure S8. ^{1}H-^{13}C HMQC spectrum of **10**.

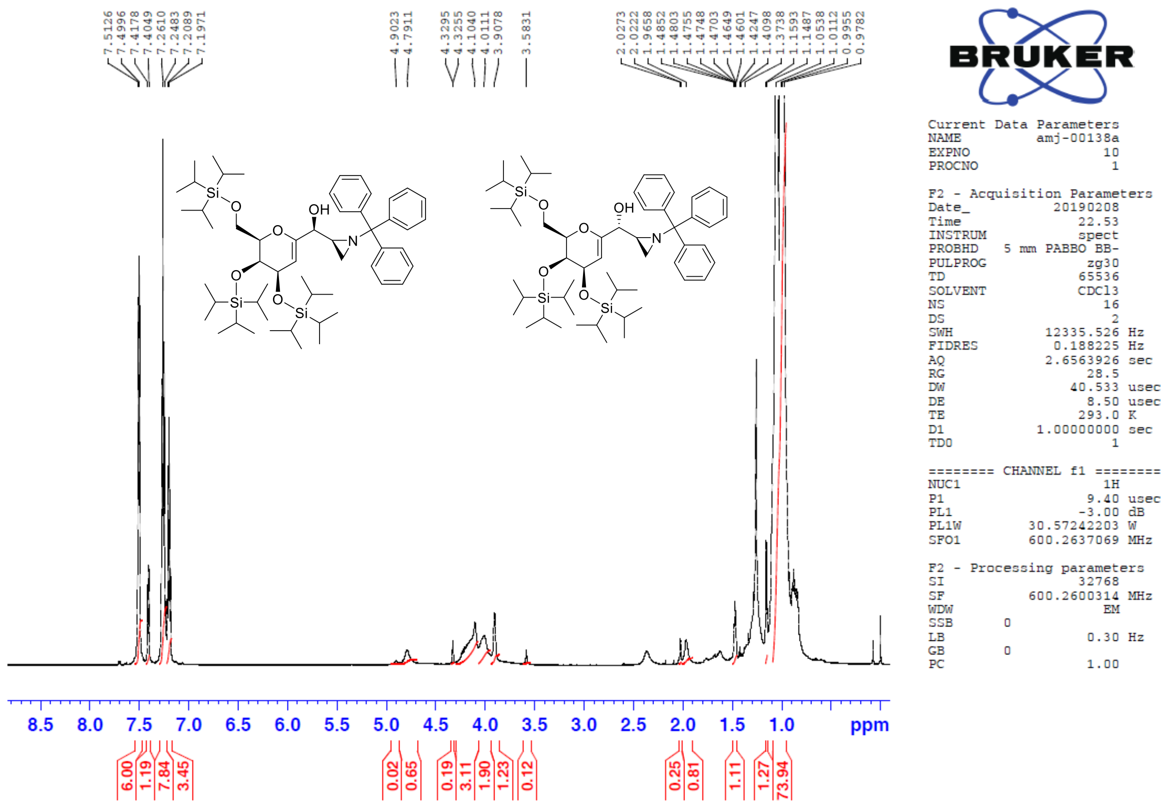

Figure S9. ^{1}H NMR (600 MHz, CD_3Cl) spectrum of **11** and **12**.

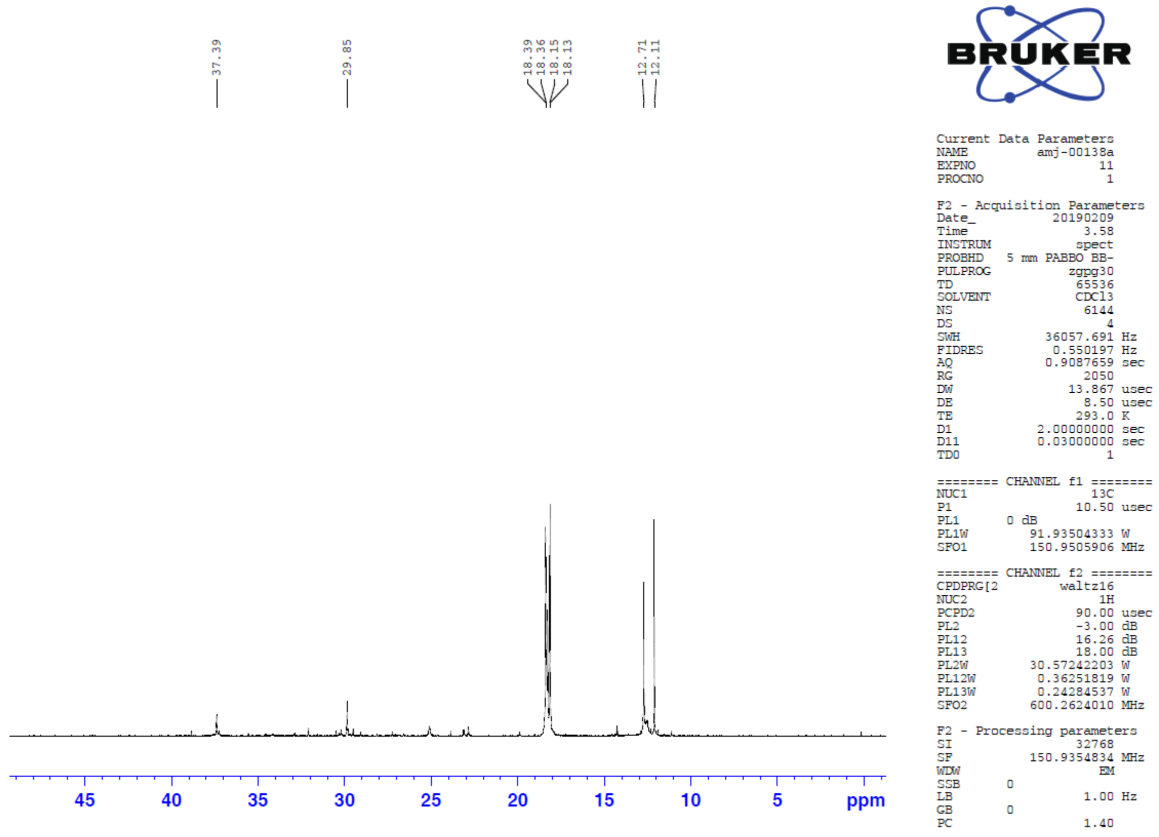

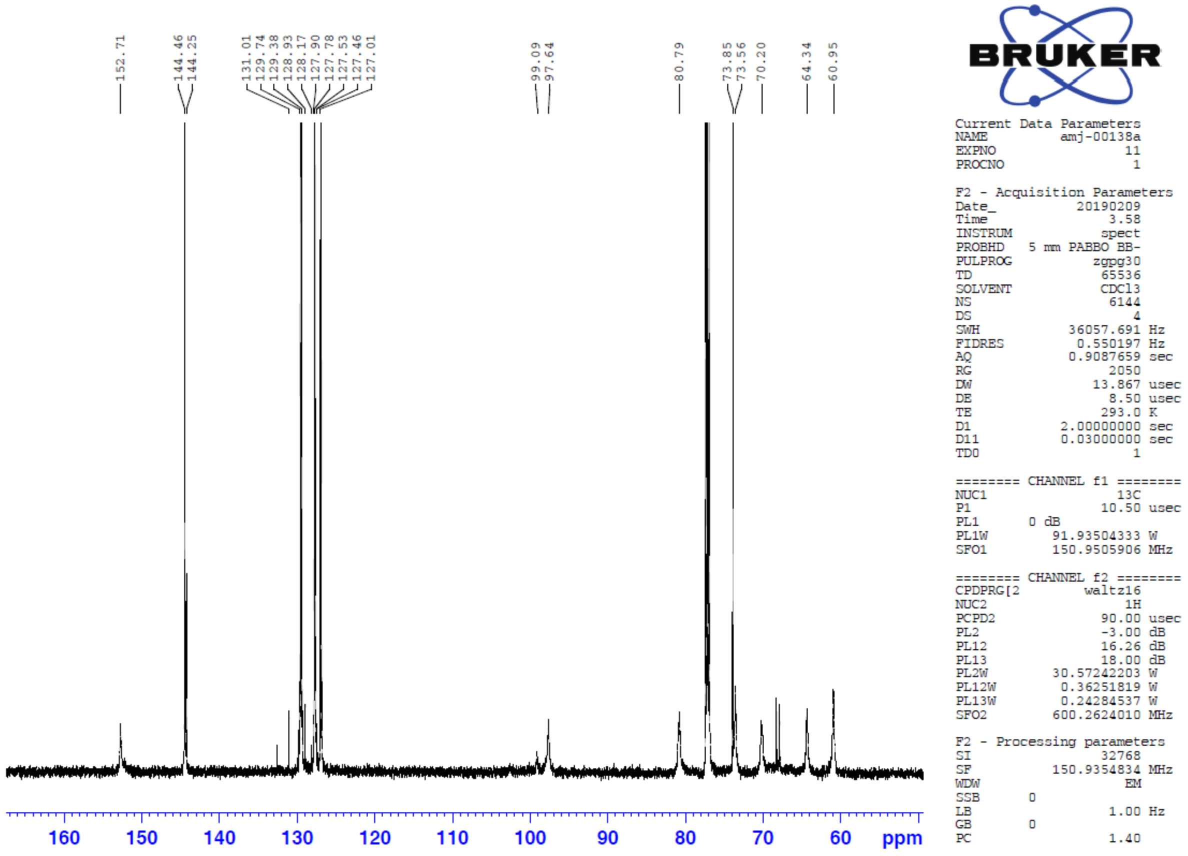

Figure S10. ^{13}C NMR (150 MHz, CD_3Cl) spectrum of **11** and **12**.

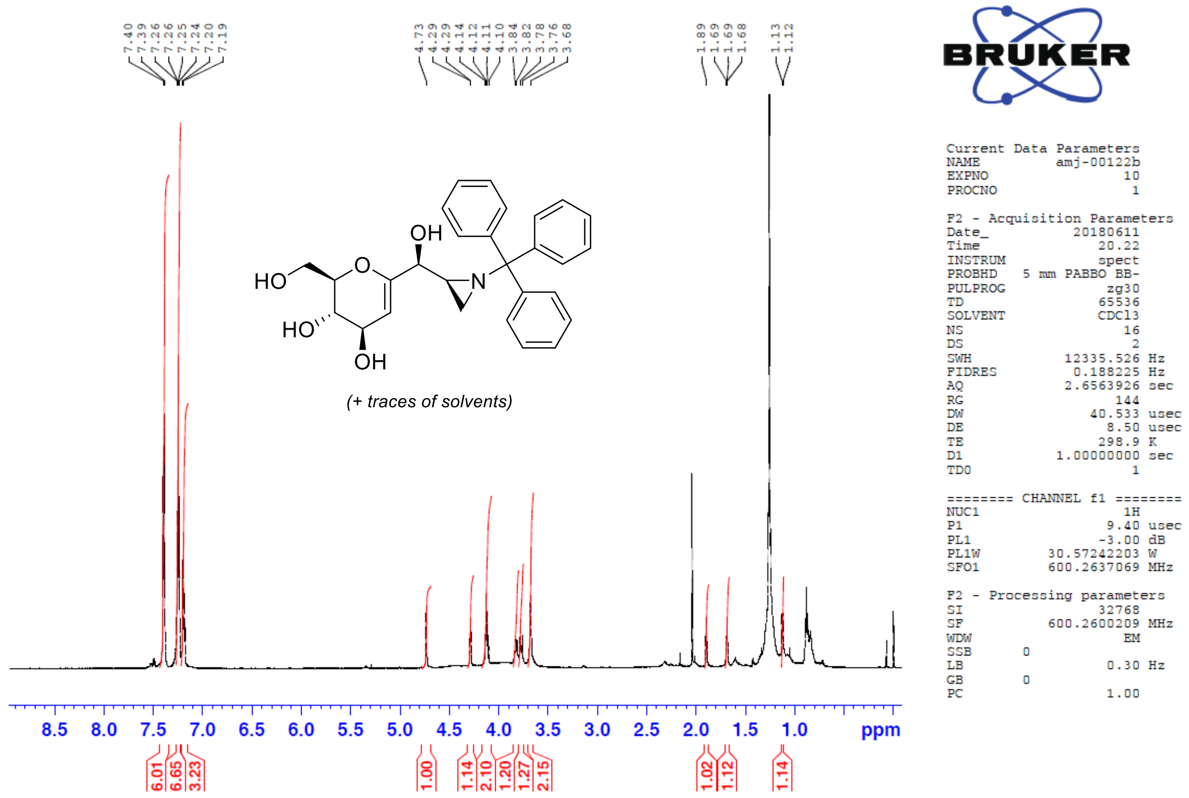

Figure S11. ^{1}H NMR (600 MHz, CD_3Cl) spectrum of **13**.

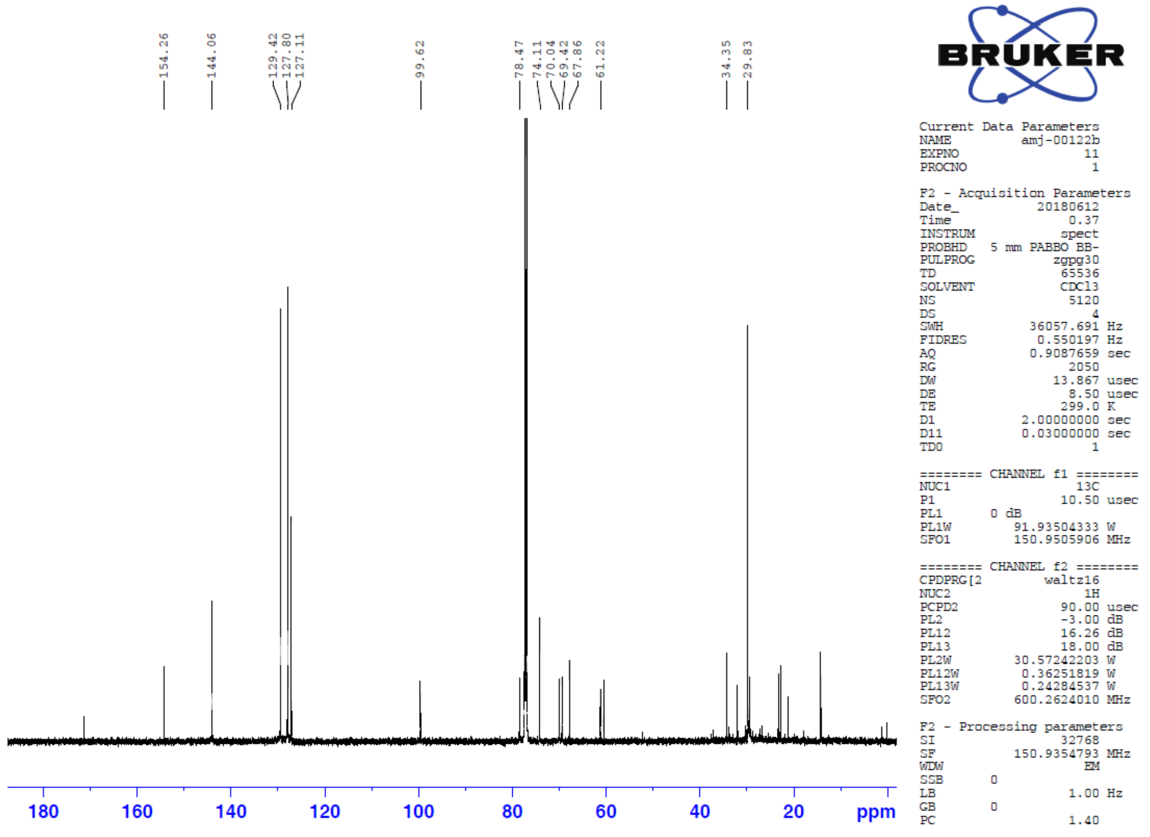

Figure S12. ^{13}C NMR (150 MHz, CD_3Cl) spectrum of **13**.

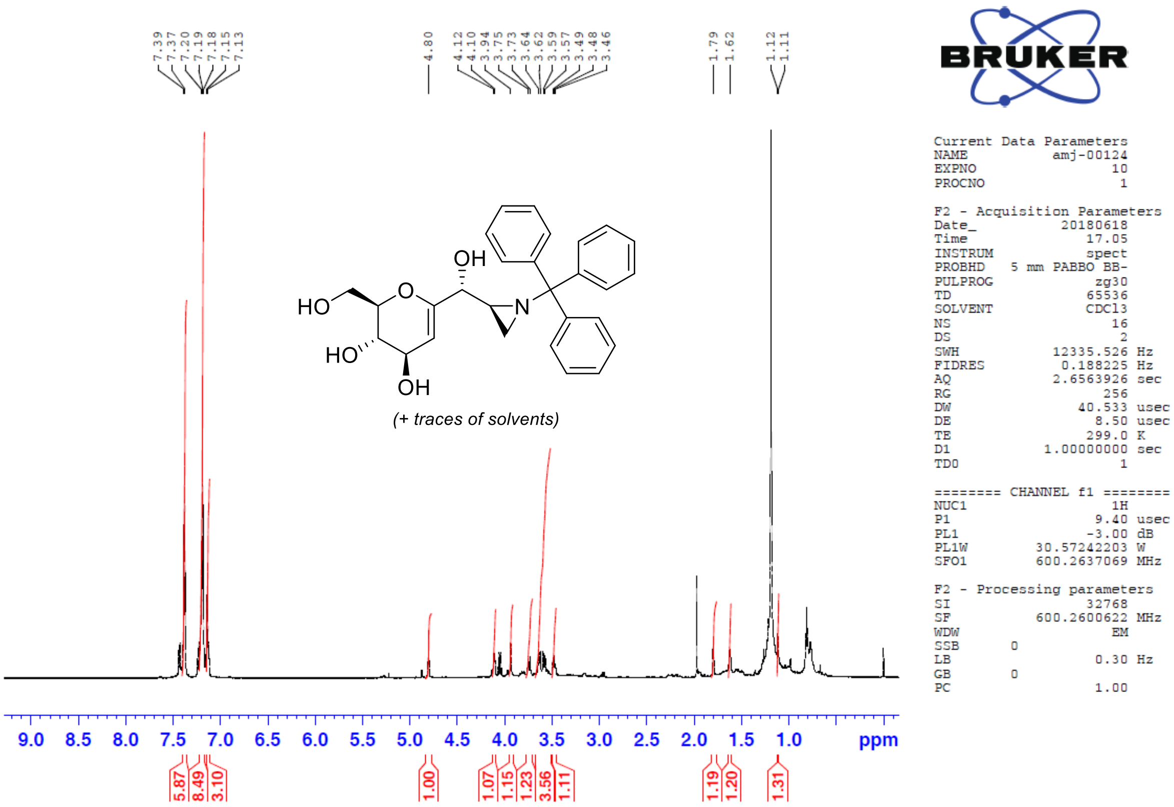

Figure S13. ^{1}H NMR (600 MHz, CD_3Cl) spectrum of **14**.

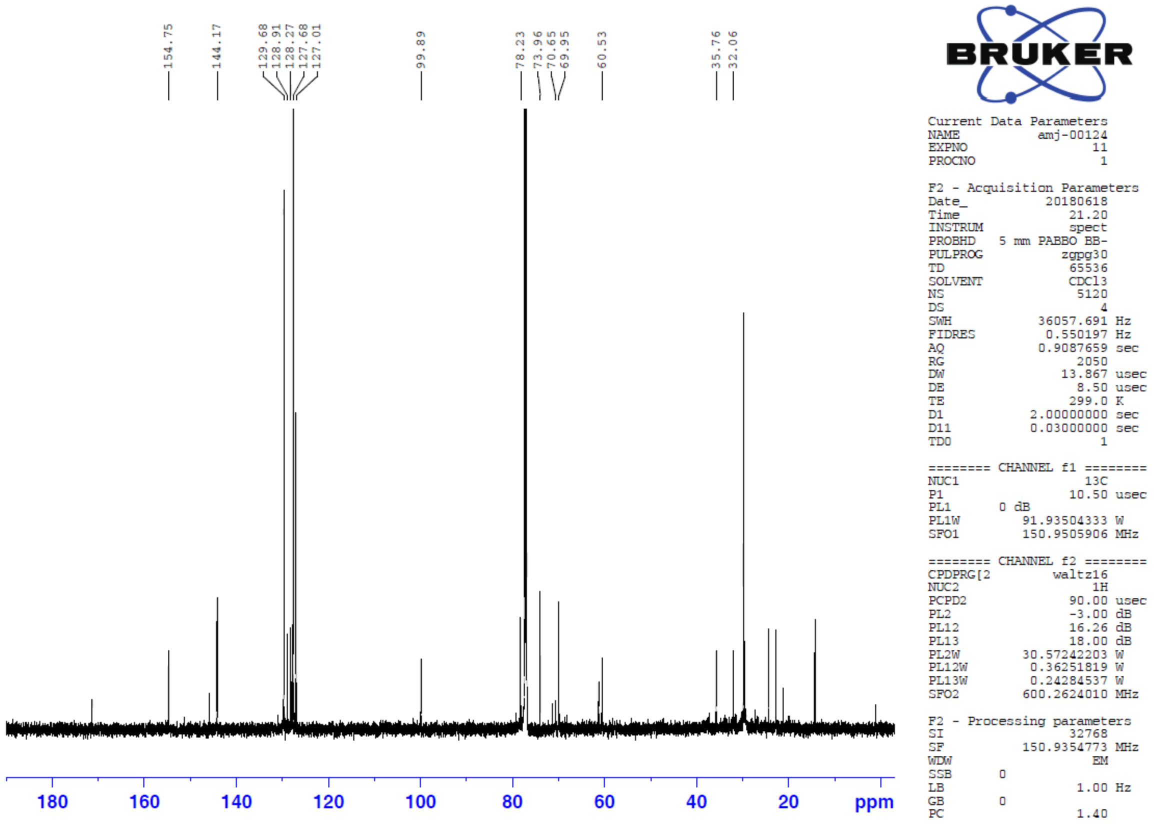

Figure S14. ^{13}C NMR (150 MHz, CD_3Cl) spectrum of **14**.

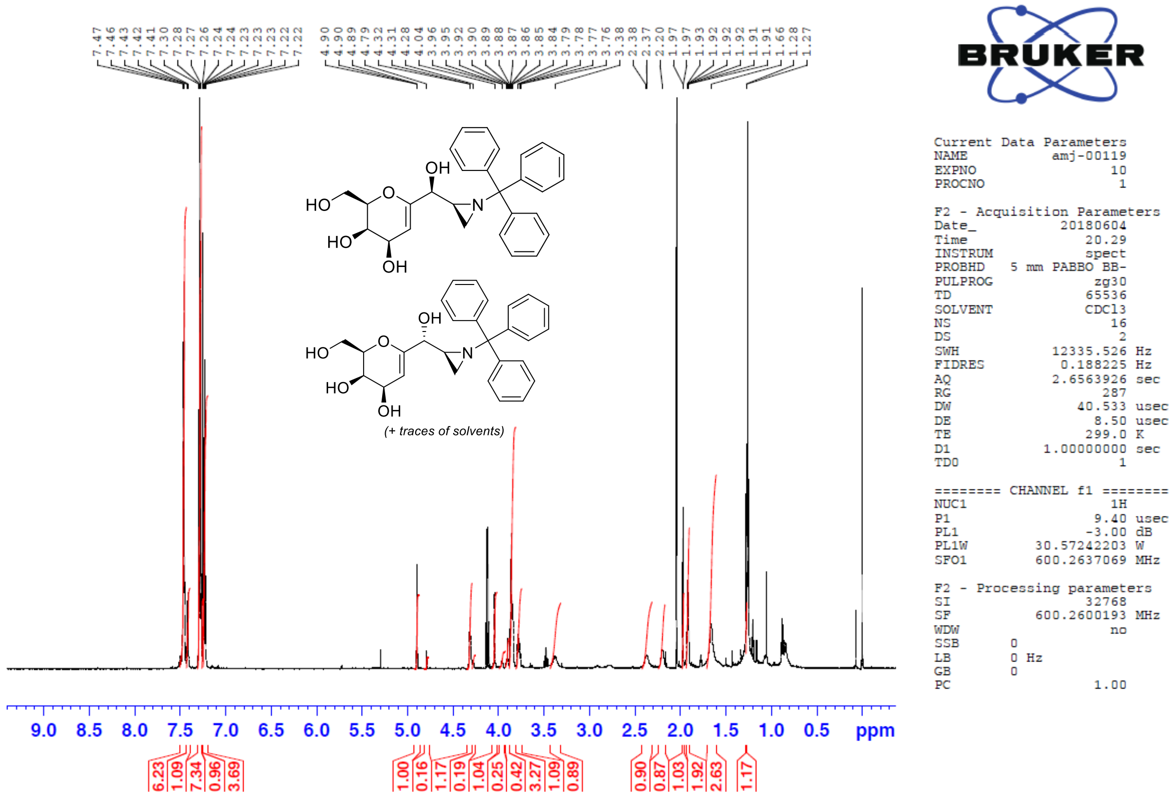

Figure S15. ^{1}H NMR (600 MHz, CD_3Cl) spectrum of **15** and **16**.

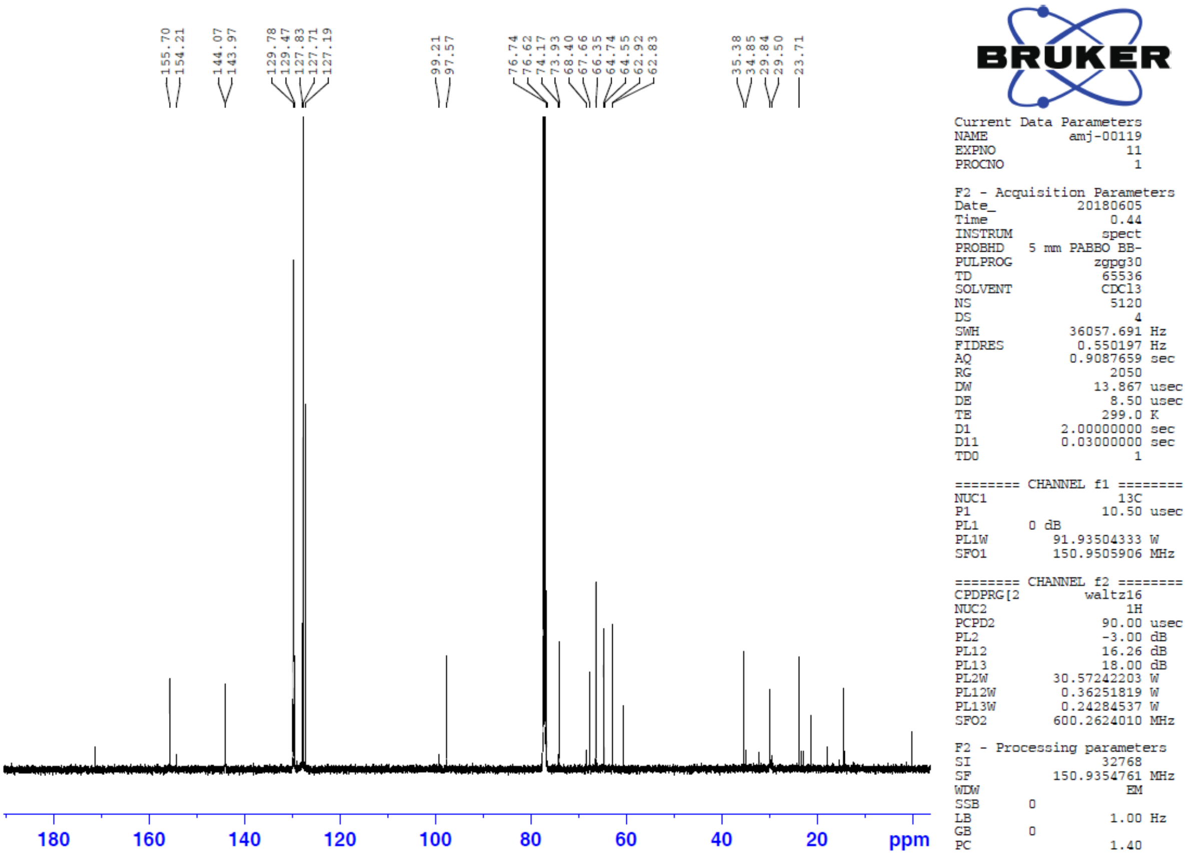

Figure S16. ^{13}C NMR (150 MHz, CD_3Cl) spectrum of **15** and **16**.

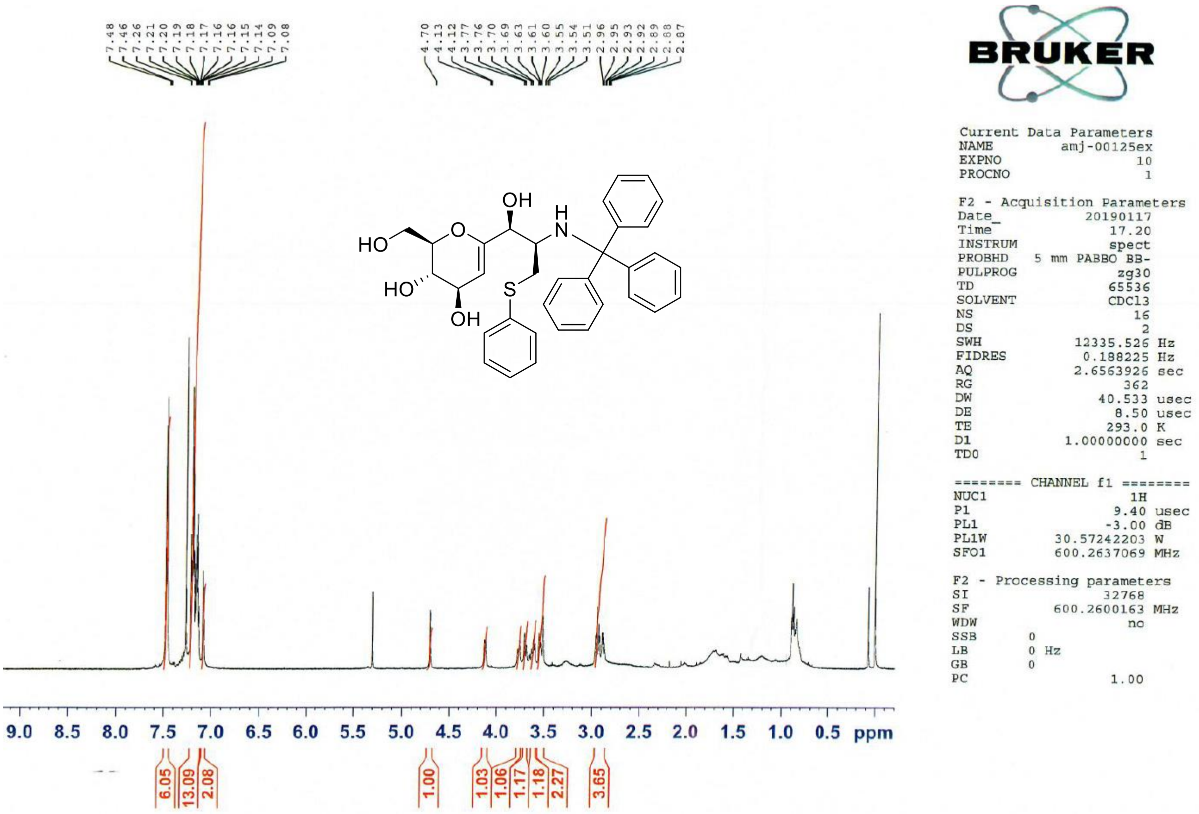

Figure S17. ^{1}H NMR (600 MHz, CD_3Cl) spectrum of **17**.

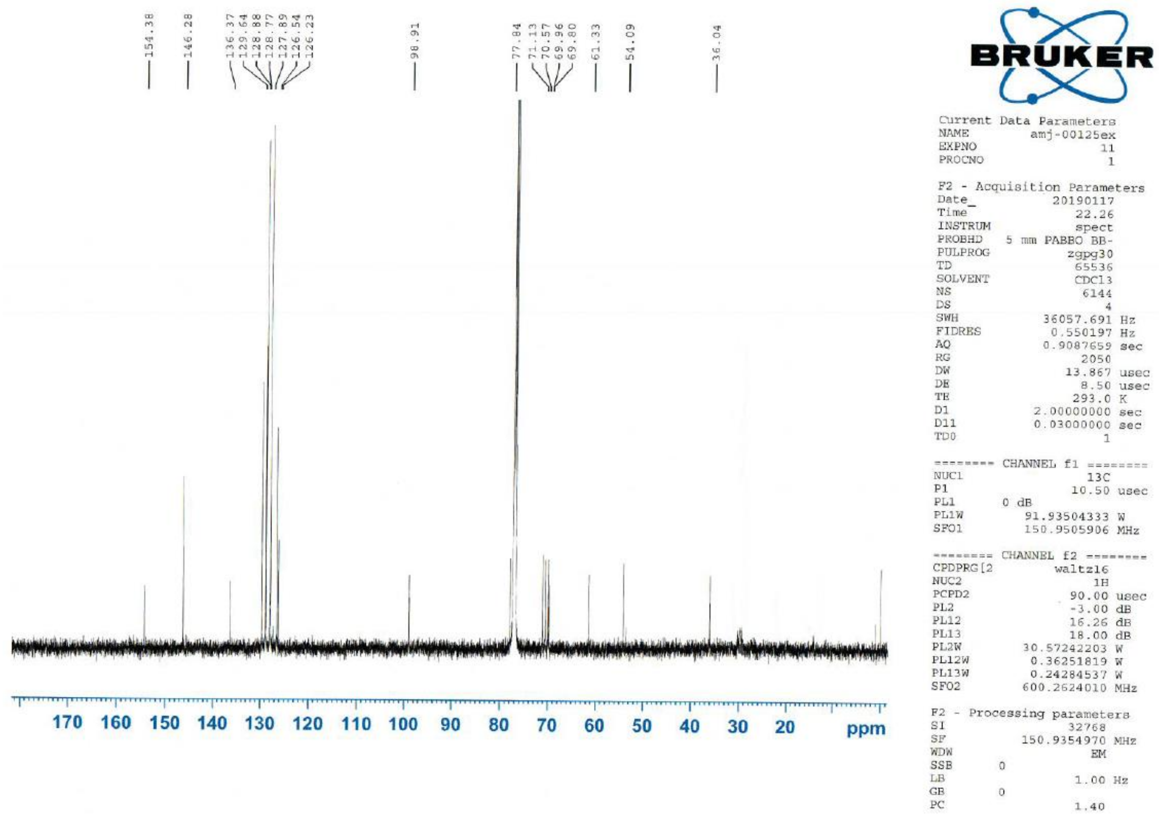

Figure S18. ^{13}C NMR (150 MHz, CD_3Cl) spectrum of **17**.

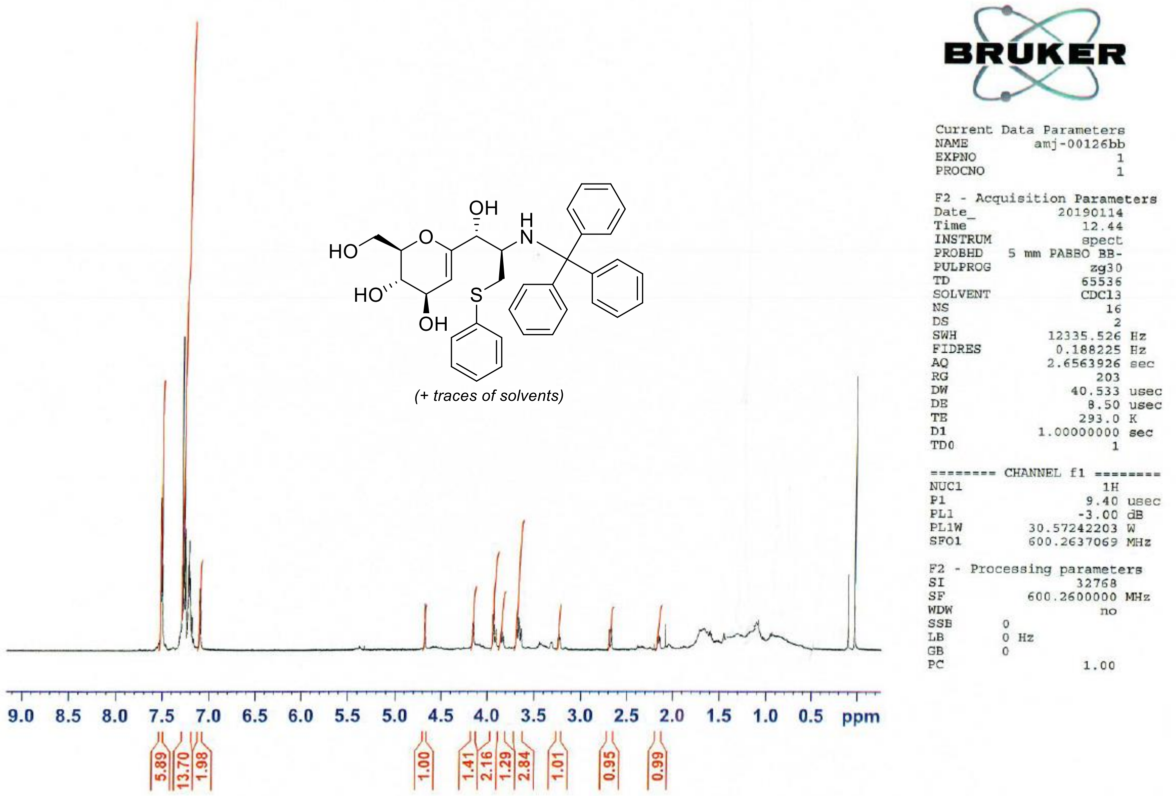

Figure S19. ^{1}H NMR (600 MHz, CD_3Cl) spectrum of **18**.

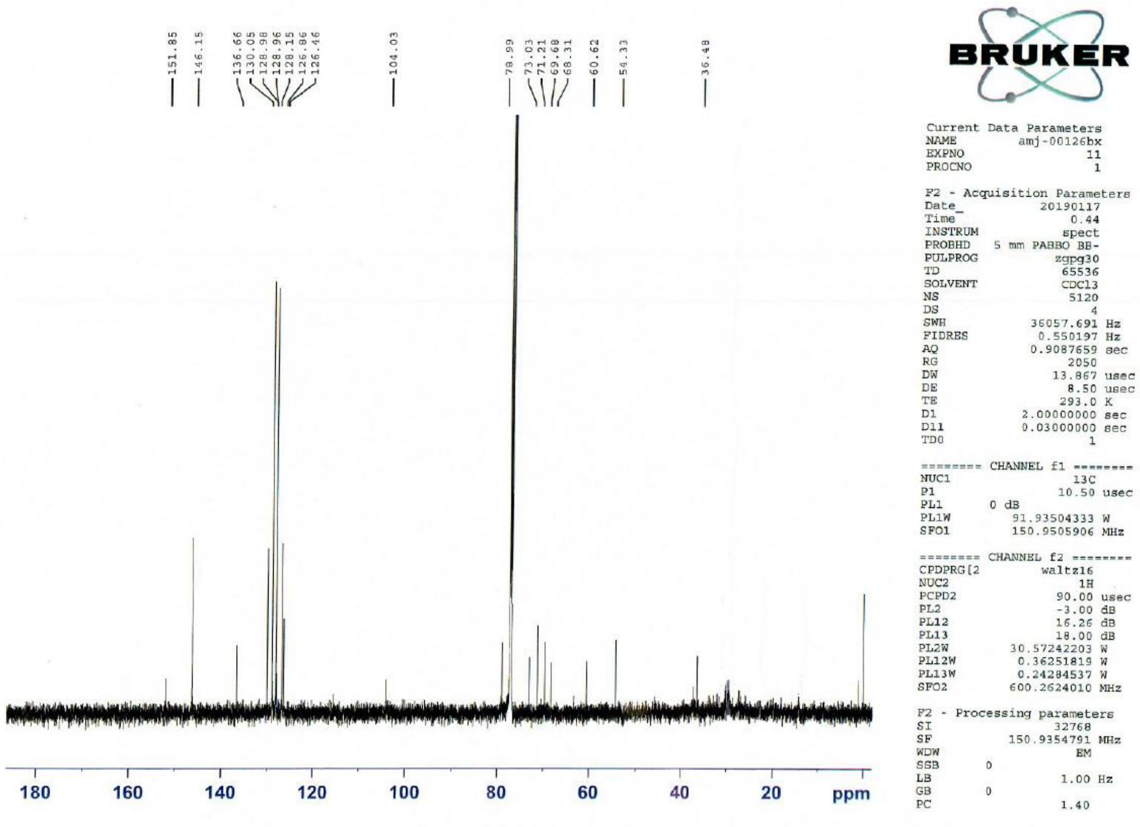

Figure S20. ^{13}C NMR (150 MHz, CD_3Cl) spectrum of **18**.

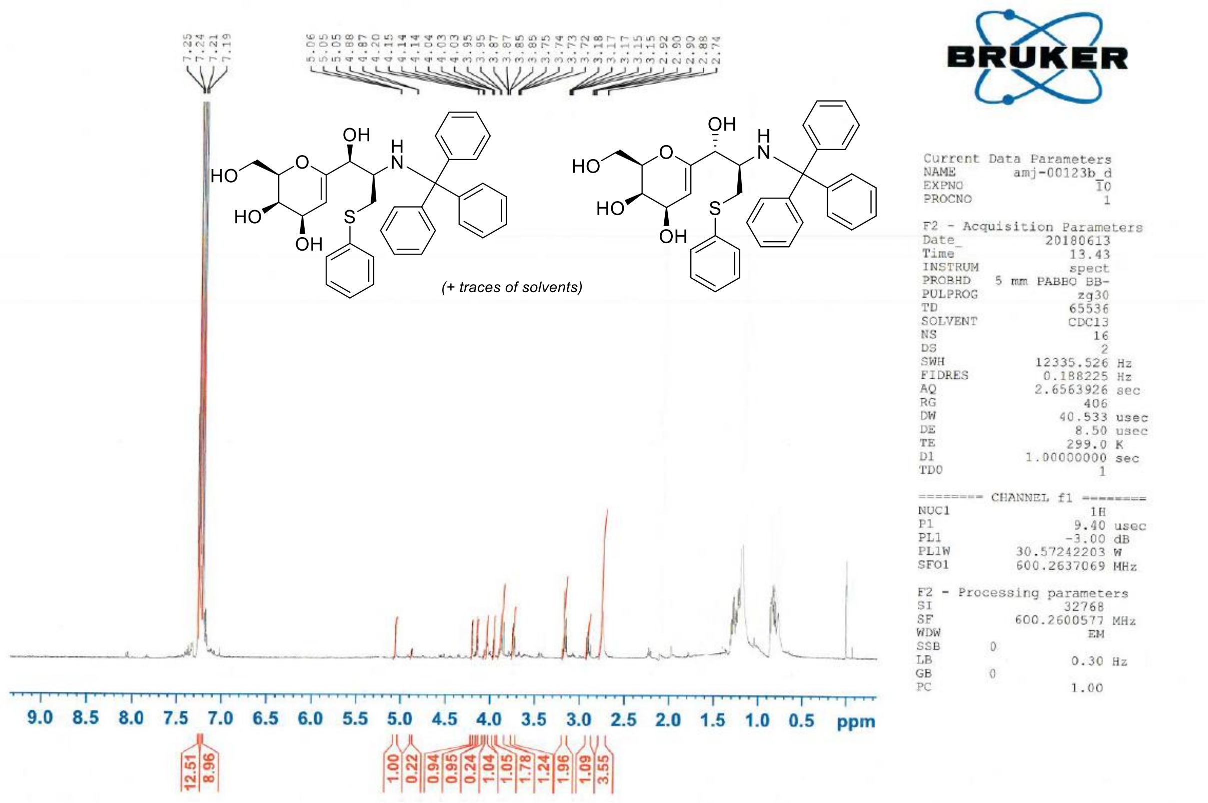

Figure S21. ^{1}H NMR (600 MHz, CD_3Cl) spectrum of **19** and **20**.

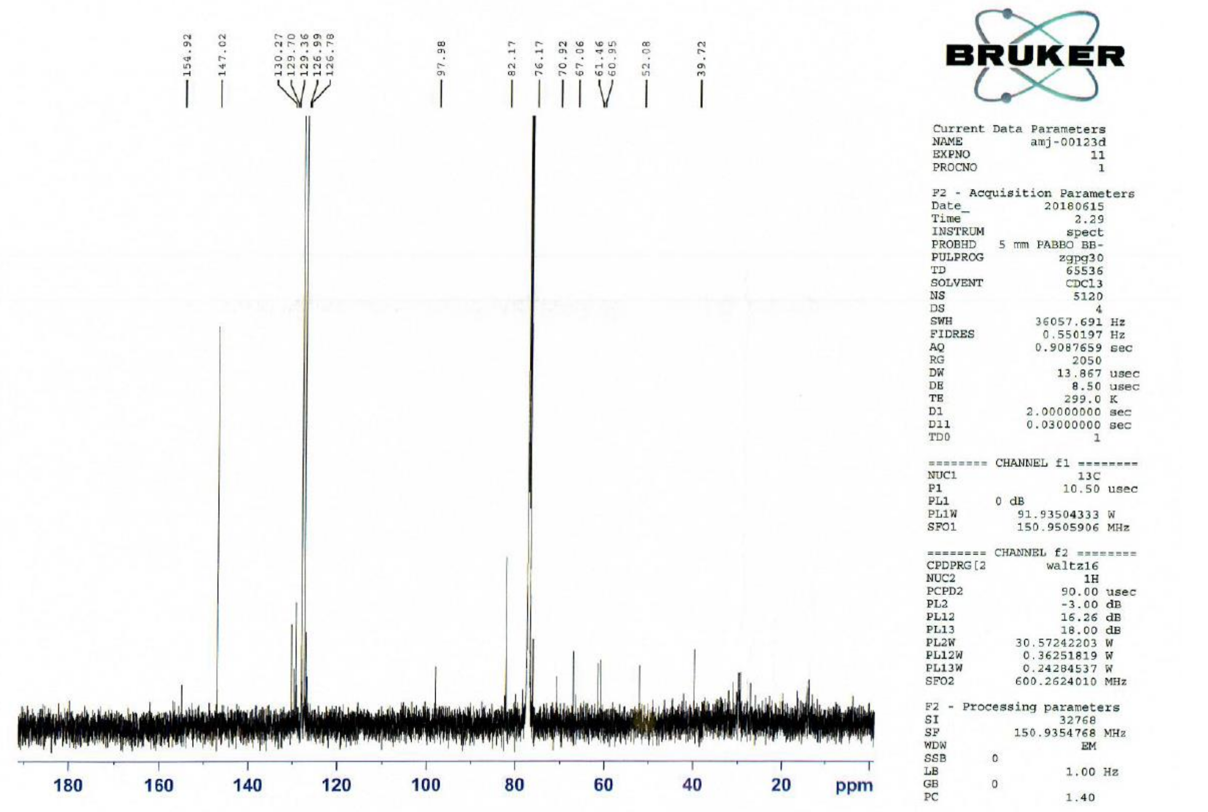

Figure S22. ^{13}C NMR (150 MHz, CD_3Cl) spectrum of **19** and **20**.
